# Potentially MOdifiable factors To ImproVe outcomes of mechanically Ventilated patients in a low-income country Intensive Care Units (MOTIVATE-ICU): rationale and protocol for a registry-embedded prospective observational study

**DOI:** 10.62675/2965-2774.20250273

**Published:** 2025-09-22

**Authors:** Cornelius Sendagire, Luigi Pisani, Alice Nuwagira, Adam Hewitt-Smith, Jane Nakibuuka, Herbert Kiwalya, Nodreen Christine Ayupo, Dominic Ogwal, Dennis Kakaire, Patience Atumanya, Betty Khainza, Hajara Nakayiza, Hawa Nakandi, Kenneth Tomanya, Martha Alupo, Lameck Ssemogerere, Erasmus Okello, Aggrey Lubikire, Andrew Kintu, Innocent Nyeko, Andrew Kamau, Chamira Kodippily, Arthur Kwizera, Abigail Beane, Rashan Haniffa, Jorge Ibrain Figueira Salluh

**Affiliations:** 1 College of Health Sciences Makerere University Kampala Uganda College of Health Sciences, Makerere University - Kampala, Uganda.; 2 Instituto D’Or de Pesquisa e Ensino Rio de Janeiro RJ Brazil Instituto D’Or de Pesquisa e Ensino - Rio de Janeiro (RJ), Brazil.; 3 Association of Anesthesiologists of Uganda Kampala Uganda Association of Anesthesiologists of Uganda - Kampala, Uganda.; 4 Uganda Heart Institute Kampala Uganda Uganda Heart Institute - Kampala, Uganda.; 5 Mahidol Oxford Research Unit Bangkok Thailand Mahidol Oxford Research Unit - Bangkok, Thailand.; 6 Mbale Regional Referral Hospital Mbale Uganda Mbale Regional Referral Hospital - Mbale, Uganda.; 7 Faculty of Health Sciences Busitema University Mbale Uganda Faculty of Health Sciences, Busitema University - Mbale, Uganda.; 8 Mulago National Specialized Hospital Kampala Uganda Mulago National Specialized Hospital - Kampala, Uganda.; 9 C-Care International Hospital Kampala Kampala Uganda C-Care International Hospital Kampala - Kampala, Uganda.; 10 Uganda Martyrs Hospital Lubaga Kampala Uganda Uganda Martyrs Hospital Lubaga - Kampala, Uganda.; 11 Kampala Hospital Limited Kampala Uganda Kampala Hospital Limited - Kampala, Uganda.; 12 Uganda Cancer Institute Kampala Uganda Uganda Cancer Institute - Kampala, Uganda.; 13 Case Hospital Kampala Kampala Uganda Case Hospital Kampala - Kampala, Uganda.; 14 Mengo Hospital Kampala Uganda Mengo Hospital - Kampala, Uganda.; 15 Platinum Hospital Uganda Kampala Uganda Platinum Hospital Uganda - Kampala, Uganda.; 16 St. Francis Hospital Nsambya Kampala Uganda St. Francis Hospital Nsambya - Kampala, Uganda.; 17 Nakasero Hospital Limited Kampala Uganda Nakasero Hospital Limited - Kampala, Uganda.; 18 TMR International Hospital Kampala Uganda TMR International Hospital - Kampala, Uganda.; 19 Kawempe National Referral Hospital Kampala Uganda Kawempe National Referral Hospital - Kampala, Uganda.; 20 Mulago Specialized Women’s and Neonatal Hospital Kampala Uganda Mulago Specialized Women’s and Neonatal Hospital - Kampala, Uganda.; 21 St. Mary’s Hospital Lacor Gulu Uganda St. Mary’s Hospital Lacor - Gulu, Uganda.; 22 Roswell Women’s and Children Hospital Kampala Uganda Roswell Women’s and Children Hospital - Kampala, Uganda.

**Keywords:** Respiration, artificial, Developing countries, Tracheostomy, Length of stay, Routinely collected health data, Registries, Critical care, Survival analysis, Intensive care units, Uganda

## Abstract

**Objective:**

To identify modifiable intensive care unit factors associated with outcomes among patients receiving invasive mechanical ventilation in a low-income setting.

**Methods:**

This prospective, multicenter, registry-embedded observational study has two components: a prospective registry-based cohort assessing patient- and care-process-related factors and a cross-sectional intensive care unit survey evaluating organizational structure. Functional intensive care units in Uganda will be included. Patients aged ≥ 15 years old requiring invasive mechanical ventilation will be enrolled. Patients extubated within 48 hours, transferred after > 24 hours, and imminent early death will be excluded. Primary outcomes will include 28-day intensive care unit mortality, intensive care unit length of stay, and mechanical ventilation duration. Tracheostomy-related outcomes will be explored in a pre-planned sub-study. Factors potentially associated with outcomes will be categorized into non-modifiable and potentially modifiable factors. Non-modifiable factors will include patient-related factors like age, comorbidities, and illness severity; potentially modifiable factors include processes of care (e.g., sedation levels) and intensive care unit organizational structure (e.g., staffing patterns). Multilevel multivariable logistic regression models will assess association outcomes. Survival analysis (Kaplan-Meier curves) will explore mortality trends. Confounders will be identified using directed acyclic graphs.

**Results (anticipated findings):**

This study will generate high-quality data on modifiable intensive care unit factors associated with ventilated patient outcomes in low-resource settings.

**Conclusion:**

This is Uganda’s first registry-embedded, multicenter intensive care unit study to systematically potentially modifiable factors associated with ventilated patient outcomes. This study will provide evidence-based insights to optimize critical care management in low- and middle-income countries by leveraging real-time intensive care unit registry data.

## INTRODUCTION

In low and middle-income countries (LMICs), up to two-thirds of intensive care unit (ICU) admissions necessitate invasive mechanical ventilation (IMV), compared to half of ICU admissions globally.^1 - 3^Though IMV is a lifesaving organ support technique, it is also linked to complications like ventilator-associated lung injury and requires complex processes of care.^4^ Despite being younger, ventilated patients in LMICs have two to four times higher mortality than high-income countries (HICs).^5 - 8^Patients with acute respiratory distress syndrome (ARDS) in LMICs face an even greater risk, 70% higher than in HICs.^9^

Several modifiable factors to improve ICU outcomes in HICs have been identified – such as adherence to low tidal volume ventilation, optimal sedation practices, fluid conservative management, and ventilator-associated pneumonia bundles – these interventions have shown significant mortality reductions and shorter ventilation durations.^1 , 10 - 13^A pooled analysis revealed that while disease severity and ventilation management were similar in both middle-income countries (MICs) and HICs, the risk of ICU mortality was 16% higher in MICs.^10^ Unfortunately, data on determinants of poor outcomes among ventilated patients in LMICs remains scarce. Yet, LMICs grapple with challenges like understaffing, limited protocol usage, suboptimal ICU processes organization, and lack of daily care plans.^10 - 16^There is a paucity of data from LMICs targeted to address these challenges; however, directly applying HIC evidence-based management strategies to LMICs is not always effective. For example, attempts to implement HIC protocols on fluid management in the African setting, in both pediatric and adult patients increased mortality contrary to expectations.^17 , 18^These studies highlight the risks of directly applying HIC protocols in LMICs without accounting for local organizational structure and preexisting care processes.

Recently, the use of critical care registries for data collection, including patient care, indicators, and outcomes, has expanded in both HICs and LMICs.^19 - 21^This streamlines research and offers cost benefits. In 2022, Uganda launched the Intensive Care Registry of Uganda (ICRU) for quality improvement and research infrastructure. This registry-embedded study aims to identify modifiable factors impacting outcomes for mechanically ventilated patients in LMICs leveraging ICRU’s data pipeline. We hypothesize that specific patient-level and organizational factors can be identified that contribute to the ICU mortality of ventilated patients in Uganda.

## METHODS AND ANALYSIS

### Study design

The Potentially MOdifiable factors To ImproVe outcomes of mechanically Ventilated patients in ICUs in a Low-income Country (MOTIVATE-ICU) study is a prospective multicentre registry-embedded observational study with two main components. The first will be a prospective observational multicenter registry-embedded study aimed at assessing patient-related and process-related factors. The second component will be a cross-sectional survey to assess the organizational structure of the ICUs included. This study protocol was registered at clinicaltrials.gov (NCT06288724). The results of the study will be reported according to the Strengthening the Reporting of Observational Studies in Epidemiology (STROBE) Statement.^22^

### Study setting

Intensive care units will be defined as geographically designated area/unit within a hospital that routinely provides IMV therapy with continuous vital sign monitoring (electrocardiographic monitoring, heart/pulse rate, noninvasive blood pressure, peripheral oxygen saturation) and designated nursing care for each bed in the unit i.e., at least three patients per week, for at least 24 hours. A descriptive study conducted in Uganda assessing the ICU capacity before the coronavirus-induced disease 2019 (COVID-19) revealed that there were 14 ICUs, with 12 being functional at the time.^15^ However, post-COVID-19, anecdotal evidence shows nearly a doubling of the ICU capacity, with nearly 25 functional ICUs to date. All ICUs currently functional in Uganda will be eligible to participate ( Table 1S - Supplementary Material ).

### Participants

Patients aged ≥ 15 years admitted to study ICUs during the study period and receiving IMV will be eligible for recruitment. We selected patients aged ≥ 15 years because, in Uganda and many LMICs, patients aged 15 years and above are typically managed in adult ICUs. Exclusion criteria include successful extubation under two calendar days, admissions for end-of-life care and/or ICU palliative support or deemed so during ICU admission, and patients transferred ≥ 24 hours after the initiation of IMV.

### Outcomes

#### Primary outcomes

The co-primary outcomes will be 28-day ICU mortality, ICU length of stay (LOS), and duration of mechanical ventilation. Table 1 details the outcome definitions.

**Table 1:** Outcomes and definitions

	Definition of measurement
Primary outcomes	
ICU mortality	Death at ICU discharge, or at 28 days after ICU admission, whichever occurs first
ICU LOS	Number of days patients spend in the ICU. Measured per episode of ICU care. Calculated using the interval (measured in hours) between the date and time of ICU admission and the date and time of ICU discharge. Rounded to the nearest 1 decimal place
Duration of mechanical ventilation	The time between endotracheal intubation and successful extubation (in case of intermittent mechanical ventilation via a tracheostomy, every day a patient needs ventilation counts as one extra day, irrespective of the duration of ventilation on that specific day). In the case of noninvasive ventilation, the duration will be assessed separately from the assessment of invasive ventilation
Secondary outcomes	
30-day hospital outcomes	Death at hospital discharge or at 30 days after ICU admission, whichever occurs first
Duration of non-pulmonary organ support	The time between the initiation of vasopressor/inotropic support or renal replacement therapy and its discontinuation must be at least 24 hours. Individual components of the composite outcome will be reported
ICU-free days	Number of in-hospital days from ICU discharge to 28 days If the patient dies after ICU discharge and before 28 days this will count as 0^(23)^
Ventilator-free days	The number of VFD-28 will be determined by subtracting the total duration of mechanical ventilation from 30 days. If a patient passes away before reaching the 28-day mark, their VFD-28 will be recorded as having zero VFD-28^(23)^
Ventilator-associated pneumonia	–Presence of fever OR altered leukocyte count PLUS –New onset of purulent endotracheal secretions OR change in sputum –WITH new and progressive or persistent infiltrate or consolidation or cavitation^(24)^ For those without a radiological diagnosis, we shall consider clinically suspected VAP
Tracheobronchitis	–Presence of fever OR altered leukocyte count (< 4 or >12 x 10^3^) –PLUS New onset of purulent endotracheal secretions OR change in sputum^(24)^
Non-infectious pulmonary complication	Clinician suspicion or radiological diagnosis of pleural effusion, atelectasis, or pneumothorax
Readmission	Unplanned ICU admission within 48 hours after ICU discharge
Unplanned extubations	Inadvertent/accidental extubations requiring reintubation
Tracheostomy related outcomes	The following outcomes will be included: –Timing of tracheostomy in terms of the number of days after intubation –decannulation rate, i.e. the proportion of patients undergoing successful decannulation during ICU stay –decannulation failure (defined as the need to recannulate for any reason between decannulation and hospital discharge) Tracheostomy-related complications: stoma infection, major bleeding, tube dislodgement, and malfunction defined as air leaks

ICU - intensive care unit; LOS - length of stay; VFD-28 - ventilator-free days at 28 days; VAP - ventilator-associated pneumonia.

#### Secondary outcomes

These will include hospital mortality censored at 30 days, duration of non-pulmonary organ support, ICU-free days, ventilator-free days at day 28, ventilator-associated pneumonia, tracheobronchitis, non-infectious pulmonary complication (clinician suspicion or radiological diagnosis of pleural effusion, atelectasis or pneumothorax), readmission, unplanned extubations. Tracheostomy-related outcomes will be explored as part of a preplanned sub-study, including tracheostomy timing, decannulation rate, decannulation failure, and complications such as stoma infection, major bleeding, tube dislodgement, and malfunction.

## Associated factors

Factors potentially associated with outcomes among mechanically ventilated patients will be analyzed as ‘non-modifiable’ factors and ‘potentially modifiable’ factors. Non-modifiable factors include patient-related factors such as age, illness severity at initiation of mechanical ventilation, comorbidities, medical *versus* surgical admission, and indication for IMV ( Table 2 ). Potentially modifiable factors will be subcategorized into care process-related factors such as Richmond Agitation Sedation Scale (RASS) targets, initial ventilator settings on day 0, use of daily spontaneous awakening and spontaneous breathing trials, use of stress ulcer and deep vein thrombosis (DVT) prophylaxis. The second subcategory will be the ICU organizational structure, which includes ICU staffing, staff-patient ratios, empowerment of non-physician staff, multidisciplinary team rounds, and protocols and checklists ( Table 2 ).

**Table 2:** Prespecified factors associated with outcomes of mechanically ventilated patients from the published literature

Patient-related factors^(1,6-9,25-29)^
1. Age (years)
2. Severity of illness score - eTropICS, APACHE II
3. Organ dysfunction score - mSOFA
4. Comorbidities - Charlson comorbidity index
5. Admission type (medical *versus* surgical)
6. Reason for ICU admission
7. Indications for IMV
8. Surgical urgency: emergency *versus* planned
9. Clinical frailty scale prior to admission
10. Readmission status
11. Immunosuppression status
**Care process factors (potentially modifiable)** ^(1,6-11,28-37)^
1. Actual RASS
2. Benzodiazepines *versus* non-benzodiazepines
3. Spontaneous awakening trial (yes/no)
4. Spontaneous breathing trial (yes/no)
5. Ventilator settings; Vt (mL/kg PBW), Inspiratory pressure on day 0
6. DVT prophylaxis
7. Stress ulcer prophylaxis
8. Head of the bed elevation
9. Vasopressor therapy and duration
10. Antimicrobial therapy duration
11. Tracheostomy timing
12. Involvement of lay caretaker
**Organizational & structural factors (potentially modifiable)** ^(11,15,30-32,38-40)^
1. ICU staffing ratios
2. Presence of trained intensivist on ward round
3. 24/7 expert intensivist coverage
4. Dedicated physiotherapist
5. Empowerment of non-physician providers
6. Protocol/checklist use
7. Number of protocols used
8. Dedicated clinical pharmacist
9. Dedicated nutritionist
10. Multidisciplinary ward rounds (yes/no)
11. Handover model (individual or MDT)

e-TropICS - Tropical Intensive Care Score; APACHE II - Acute Physiology and Chronic Health Evaluation II; mSOFA - modified Sequential Organ Function Assessment; ICU - intensive care unit; IMV - invasive mechanical ventilation; RASS - Richmond Agitation Sedation Scale; Vt - tidal volume; PBW - predicted body weight; DVT - deep venous thrombosis; MDT - multidisciplinary team.

## Data collection

**Figure 1:**
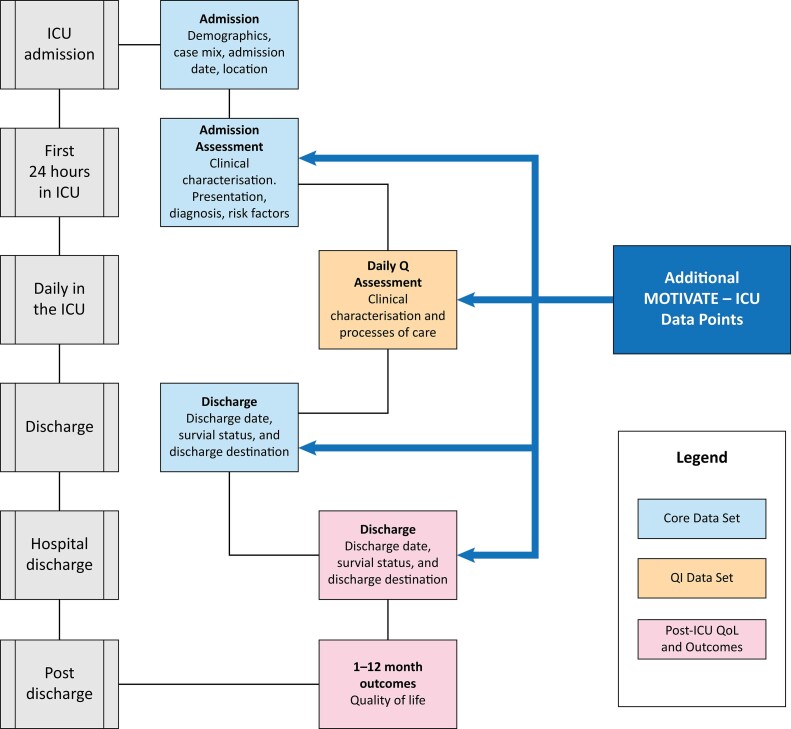
Integration of the MOTIVATE-ICU Electronic Case Report Form in the Intensive Care Registry of Uganda registry Case Report Form. ICU - intensive care unit; Q - Quality; QI - Quality Improvement; QoL - Quality of Life.

To ensure high-quality and standardized data collection, the following procedures will be implemented:

1.Direct Electronic Data Entry:

–Patient-level data will be entered directly into the PROTECT cloud-based ICU registry platform, which is managed by the ICRU, via a laptop or tablet ( Figure 1 ).

–This platform enables real-time data capture and seamlessly integrates with existing ICU records.

2.The ICRU registry collects patient-level data, including core ICU admission data, daily ICU care variables, and outcomes at ICU discharge ( Tables 2SA and 2SB - Supplementary Material ). Illness severity estimation already exists in the platform using both the APACHE II and the Tropical Intensive Care Score (e-TropICS).^26^

3.Registry-Embedded Data Collection for MOTIVATE-ICU:

–The MOTIVATE-ICU study variables are fully integrated within the ICRU as an additional data layer.

–Additional variables captured in this study include clinical frailty scale,^41^ ventilator settings, tracheostomy data, sedation care processes, fluid monitoring, primary indication for IMV, and related complications. To assess organ dysfunction, we shall use the modified Sequential Organ Function Assessment score (mSOFA)^27^ by embedding urine output into the core ICU admission data ( Table 3S - Supplementary Material ).

–These variables are recorded prospectively within the ICRU framework to maintain consistency and minimize duplication.

4.Data extraction and quality assurance:

–Data extraction will occur directly from the ICRU platform. The platform operates on a standard data model, enabling standardized case-mix reporting, management insights, and real-time analytics ( Figure 1 ).

–To ensure data integrity, periodic validation checks, audit trails, and cross-verification procedures will be conducted.

5.ICU organizational and process data collection:

–Hospital and ICU-level characteristics will be collected at each site via an interviewer-administered questionnaire ( Table 4S - Supplementary Material ) with the ICU director and/or in-charge nurse.

–Variables of interest include ICU type (medical-surgical *versus* specialty), Presence of critical care training programs, ICU admission volumes (preceding year), staffing patterns and multidisciplinary team presence, and organizational and care process characteristics.

6.Table 2SA and 2SB ( Supplementary Material ) details a complete list of ICU patient-level variables.

## Follow-up

Given the potential burden of data collection, the patient recruitment period in each participating ICU will be a minimum of 3 months up to a maximum of six months, with a follow-up period of up to 30 days in-hospital or until hospital discharge, whichever comes first.

## Sample size

We aim to include all consecutive patients admitted to participating ICUs to reduce potential selection bias and minimize the temporal variation of care processes. Based on aggregated data (published and unpublished) from LMICs including Uganda’s ICU cohorts^2 , 10 , 12 , 28 - 32 , 38^ the lowest measured mortality among patients receiving IMV was approximately 40%. A total of 250 events (deaths) would allow us to evaluate at least 25 associated variables in multivariable models. We will therefore target to enroll 625 patients receiving IMV.

## Procedures to ensure data quality

The following site-level procedures will be followed:

- To ensure standardization of the study procedures, each site shall have a site lead and dedicated data entrant(s) responsible for data collection while using the ICU registry platform. Before beginning recruitment, each site lead and data entrant(s) will receive training.
- Each site will have a site lead responsible for weekly validation of patient episodes regarding completion and data quality.
- Each site will have IT support and participate in monthly meetings to dispel doubts and solve potential challenges related to data entry.

## Handling of missing data

All data pertinent to the study objectives will be mandated for all study ICUs during the study period. The study platform will flag missing data to the data collector in case of incompleteness. All sites will be monitored weekly to ensure the validation of patients entered and the completeness and accuracy of the data to ensure there is no missing data. This is being a prospective registry-embedded study, we expect a low percentage of missing data pertinent to the primary objectives of the study.

## Statistical analysis

**Descriptive statistics:** ICU and patient characteristics will be summarized using appropriate descriptive statistics; for continuous variables, reported as mean ± standard deviation (SD) or median (interquartile range [IQR]) based on their distribution, and for categorical variables, summarized as counts (n) and percentages (%). Statistical tests will be applied appropriately for univariate associations between patient-, care process-, and ICU-level factors and the primary outcomes (ICU mortality, ICU LOS, and mechanical ventilation duration). For normally distributed continuous variables, we shall use the Student’s t-test (for two groups) and analysis of variance (ANOVA) (for ≥ 3 groups), while for non-normally distributed continuous variables, we shall use the Mann-Whitney U test (for two groups) and Kruskal-Wallis test (for ≥ 3 groups). For categorical variables, we shall use the Chi-square test (if the expected counts are ≥ 5 in all cells) and Fisher’s exact test (for small sample sizes). Eligible variables include patient-related, care process and organizational factors potentially associated with ICU mortality, LOS, and mechanical ventilation duration ( Table 2 ). According to Marshall et al. ( Table 5SA - Supplementary Material ), we shall classify ICUs based on the proposed classification of ICUs.^42^ These analyses will inform the multivariable modeling approach.

**Primary outcomes & multivariable models:** we shall have the following co-primary outcomes for the study: for ICU mortality, binary outcome: alive *versus* dead; for ICU LOS, continuous, right-skewed; for mechanical ventilation duration, continuous, right-skewed. We shall use pre-specified multilevel models to assess the association between patient-related-, care process-related-, and ICU organizational factors and patient outcomes. For ICU mortality (primary outcome), we shall use multilevel logistic regression (Random-Intercept Model):

–Level 1 (patient-level factors) including age, e-TropICS, mSOFA, Charlson comorbidity index, clinical frailty scale, reason for admission, admission type, surgical urgency, immunosuppression. In addition, care process-related factors – potentially modifiable: sedation practices: deep sedation as percentage of patient-days, sedative medication (benzodiazepines *versus* non-benzos); ventilation strategies: spontaneous awakening trial (percentage of patient-days), spontaneous breathing trial (percentage of patient-days), ventilator settings (median tidal volume [mL/kg predicted body weight], median inspiratory pressure, median positive end-expiratory pressure); preventive and supportive care: DVT prophylaxis (percentage of patient-days), stress ulcer prophylaxis (percentage of patient-days), head-of-bed elevation (percentage of patient-days); organ support measures: vasopressor therapy duration (median time), antimicrobial therapy duration (median time), tracheostomy timing (median time), cumulative fluid balance (median); lay-care provider presence: (yes/no).

–Level 2 (ICU-level organizational factors – potentially modifiable); ICU staffing ratios, presence of 24/7 intensivist, multidisciplinary rounds, presence of a clinical pharmacist, physiotherapist, nutritionist, use of care protocols.

–ICU site as a random intercept to account for clustering effects based on the classification listed in table 5SB ( Supplementary Material ).

We shall use the above factors for ICU LOS and mechanical ventilation duration. For ICU LOS) we shall use the negative binomial regression (chosen due to overdispersion in count data), and for mechanical ventilation duration, we shall use the Cox Proportional Hazards Model, with ICU discharge and death as competing risks.

In addition to the primary outcomes, we will conduct secondary outcome analyses to assess the impact of patient-related, care process-related, and ICU organizational factors on key clinical and process-related outcomes. These secondary outcomes include duration of non-pulmonary organ support, hospital mortality (censored at 30 days from ICU admission), ICU-free days, ventilator-free days, ventilator-associated pneumonia, tracheobronchitis, non-infectious pulmonary complications, readmission, unplanned extubations, incidence of pressure sores, and tracheostomy-related outcomes.

**Confounder selection using directed acyclic graphs:** to ensure proper confounder control and avoid overfitting, we will construct a directed acyclic graph (DAG) to identify the minimum adjustment set necessary for estimating the effect of patient-related factors, ICU organizational and care process factors on patient outcomes. The DAG will be based on prior literature on ICU outcomes and modifiable factors in LMICs, expert consensus from intensivists and methodologists, preliminary analysis of data distribution, and collinearity among predictors. Confounders identified via DAG analysis will be retained in all models regardless of statistical significance, ensuring robust estimation of patient-related factors, ICU care processes, and organizational factors on mortality and other outcomes.

**Handling of missing data:** multiple imputation with chain equations will be used to address missing data in key variables. Sensitivity analysis will compare: complete case analysis *versus* imputed datasets and exclusion of ICUs with > 30% missing data. False discovery rate correction will be applied for subgroup analyses to control for multiple comparisons.

All statistical analyses will be conducted using R (http://www.r-project.org) and Statistical Package for the Social Sciences (SPSS) 21 (IBM Corp.), which may be used for additional descriptive analyses. The final detailed statistical analysis plan will be available on the ClinicalTrials.gov (NCT0628872) record before the database is locked.

## Ethical approval and consent to participate

This study will be conducted according to the principles of the Declaration of Helsinki (revision Fortaleza, Brazil, October 2013). Ethical approval was obtained from the Uganda Heart Institute Research and Ethics Committee (UHIREC-0009), and 15 hospital administrative approvals were in place at the time of this writing.

We shall obtain deferred consent from the legal representative within 72 hours of recruitment into the study since most ICU admissions are emergency admissions, and informed consent during the initial 48 hours of admission may not be possible. In addition, without exception, patients admitted to ICU for ventilator support are unable to give informed consent. Persons who may take the role of legal representative following the Medical Treatment Agreement Act (WGBO) are: a predefined representative, husband or wife, registered partner or other life partner, a parent or child, brother or sister, and incidentally a curator appointed by a judge. For patients with no legal representative and are unable to give informed consent during in-hospital stay, we were granted a waiver of informed consent. The experience of ICU patients enrolled under deferred consent is mainly positive, as shown in the NICE-SUGAR trial, in which participants were included using deferred consent.^43^ Most of the patients were happy with the decision made by the representative (93%) and would have granted consent if asked (96%).^44^

Oral consent from each ICU director and/or in-charge nurse will be sought to conduct the interviewer-administered survey.

## Dissemination

The study results will be submitted for publication regardless of the results after completion and analysis. We shall make the study findings widely available and disseminate them through conferences and peer-reviewed journals.

## Data sharing

The authors encourage interested parties to contact the corresponding author with data sharing requests, including requests for access to additional unpublished data. The Intensive Care Registry of Uganda is part of a larger network that has appointed a Data Access Committee (DAC). Curated data may be shared with third parties for research purposes following written approval from the DAC.

## Discussion and study status

The MOTIVATE-ICU registry-embedded study aims to identify potentially modifiable factors associated with outcomes among invasively ventilated patients in a low-income country.

As ICU registries are expanding in LMICs, it is pivotal understanding the practical outputs of such data collection systems and how the data generated can be leveraged to improve patient care. The ICRU was initiated in 2020 and uses a cloud-based platform (PROTECT-ICU), a standard data model, and standardized nomenclature to ensure interoperability of reporting between local, national, and other international stakeholders. When writing this, 8 of Uganda’s 25 ICUs were part of the ICRU, and approximately 5,000 patients were therein. Leveraging this, we embedded the MOTIVATE-ICU study into the registry by adding extra necessary variables ( Tables 2SA and 2SB - Supplementary Material ) relevant to address the objectives.

According to the Donabedian model, improving the quality of healthcare and improving outcomes of interest, rests on three components; the structure, care processes, and outcomes.^45^ Various studies from HICs on ICU organization structure, care bundles, and protocols have shown a significant impact on the reduction of mortality, duration of ventilation, and ICU length of stay, albeit with some inconsistencies.^6 , 28 - 30 , 38^ Based on what we currently know from the ORCHESTRA study done in Brazil, an upper middle-income country (UMIC), organizational factors, including protocol implementation, are the key targets to improve ICU outcomes and efficiency.^31 , 32^There has been considerable data describing the physical structural set-up and staffing of ICUs in LMICs.^15 , 40 , 46 , 47^ However, there is limited data on organizational features and care processes and their impact on outcomes in lower-income settings like Uganda.

Directly implementing evidence-based care processes from higher income settings may not yield similar results when implemented in resource-poor settings. For example, two African studies that implemented fluid therapy as per protocols used in HICs revealed more harm than benefit in both critically ill adults and pediatric populations.^( 17 , 18 )^ For other processes, implementation in LMICs has improved consistently, but outcomes remain lagging compared to HICs, which are probably affected by many factors. For instance, the use of protective mechanical ventilation in patients without ARDS was shown to be comparable between MICs and HICS during the first days of ventilation, but with a significant difference in ICU mortality.^10^ The same was seen for ventilation in ARDS patients, albeit with a slight difference in protective ventilation implementation.^( 1 , 6 )^

One of the key potential interventions likely to show impact in low-resource settings compared to HICs is tracheostomy placement and timing. Tracheostomy timing, while improving ICU length of stay and duration of mechanical ventilation, has not impacted mortality in HICs,^33 - 35^ but may have a significant impact in a setting with limited resources. A preplanned sub-study of MOTIVATE-ICU will explore timing, complications, and outcomes in patients undergoing tracheostomy in a low-income country like Uganda, complementing data from LMICs from other African countries.^36 , 37^

The strengths of the current study are its prospective design, registry-embedded nature, and multicenter inclusion. Integrating this study with the ongoing national ICU registry is a significant strength. This approach ensures comprehensive and high-quality data collection and demonstrates the feasibility of embedding research within existing clinical infrastructure in resource-limited settings. By leveraging the established registry platform, the study minimizes operational costs, enhances data accessibility, and improves scalability. This model provides a practical framework for other LMICs to develop similar research initiatives by utilizing their existing registries or creating adaptable systems tailored to their healthcare contexts. Such integration has the potential to facilitate regional collaborations, foster data-driven quality improvement programs, and standardize critical care practices across diverse settings.

This study has several potential limitations. While the study’s findings will be highly relevant to low-income ICU settings, several limitations must be acknowledged. Variations in documentation quality across participating ICUs may affect data completeness and accuracy. To mitigate this, we will implement standardized data entry protocols, periodic audits, and cross-validation checks between registry data and source documents to enhance reliability. To assess the robustness of our findings, we will perform sensitivity analyses, such as excluding sites with higher proportions of missing data, conducting subgroup analyses by ICU level, and using multiple imputation techniques for incomplete records where appropriate.

Recognizing that findings from Uganda may not be directly transferable to all LMICs, we will compare key study outcomes with existing ICU studies from other African and LMIC settings. Additionally, by aligning our data variables with global critical care registries, our findings can inform broader discussions on ICU care in resource-limited settings beyond Uganda. These strategies will enhance the study’s internal validity while strengthening its external applicability.

The study’s findings, especially those related to organizational structure and process-related factors, may be specific to the context of low-income countries and thus may not be generalizable to settings with different healthcare systems, resources, and ICU capabilities. The cross-sectional survey assessing the organizational structure of ICUs relies on self-reported data, which can introduce biases and inaccuracies.

## SUPPLEMENTARY MATERIAL

**Table 1S:** List of current intensive care units in Uganda - 2023

Hospital	ICU type	Location	#ICU beds	#Admissions/month
Mulago NRH	GICU	Kampala	27	20
Mbarara RRH	GICU	Mbarara	8	12
Lubaga Hospital	GICU	Kampala	4	10
Mbale Regional Referral Hospital	GICU	Mbale	5	5
Kiruddu NRH	MICU	Kampala	4	8
Nakasero Hospital	GICU	Kampala	8	15
Uganda Heart Institute	CVICU	Kampala	4	8
Kampala Hospital	GICU	Kampala	6	8
Nsambya Hospital	GICU	Kampala	5	8
Case Hospital	GICU	Kampala	9	8
Medipal International Hospital	GICU	Kampala	10	5
Medical Hub	GICU	Kampala	4	4
TMR International Hospital	GICU	Kampala	9	10
Cure Hospital Kampala	Neuro-ICU	Mbale	5	8
Lacor Hospital	GICU	Gulu	8	12
UMC Victoria Hospital	GICU	Kampala	5	8
Platinum Hospital	GICU	Kampala	4	6
International Hospital Kampala	GICU	Kampala	11	12
Kawempe NRH	Obs ICU	Kampala	5	8
Mengo Hospital	GICU	Kampala	4	6
Norvik Hospital	GICU	Kampala	4	4

ICU - intensive care unit; NRH - national referral hospital; GICU - general intensive care unit; RRH - regional referral hospital; MICU - medical intensive care unit; MSWNH - Mulago Specialized Womens and Neonatal Hospital; CVICU - cardiovascular intensive care unit.

This is an open access article under the CC BY license ( https://creativecommons.org/licenses/by/4.0/ ).

**MOTIVATE-ICU eCRF ICU registry platform variables**

**Data points - MOTIVATE additions in blue; ICRU in black**

**Core variables**

These variables provide essential information to enable reporting of case mix, acuity, and risk adjusted outcome. The patient identifiers are used to enable individual intensive care unit (ICU) stakeholders to track patients during the encounter. These are removed during data curation and an encounter ID is generated. Variables in bold are the parent variable, subsequent non bold variables provide greater granularity.

**Table 2SA:** Admission data

Variable name	Data entry format	Definitions for collection
Study ID ***	Alphanumeric	Automatically generated based on study site
Medical record number ID ***	Alphanumeric	Hospital number given to the patient for specific hospital encounters or repeated encounters
Age *P**	Years	Chronological age on the day of ICU admission
Sex *P**	Options: male, female, intersex	Sex at birth
Contact number *ID* †	Numerical	Phone number of patient or NOK that can be used to contact
Date of hospital admission *P**	yyyy/mm/dd	Date of arrival in the hospital
Time of hospital admission *P**	hh:mm:ss	Time of arrival in the hospital
Date of ICU admission *P**	yyyy/mm/dd	Date of arrival in the ICU
Time of ICU admission *P**	hh:mm:ss	Time of arrival in the ICU
ICU admission source *P**	Dropdown list	Patient’s clinical location prior to ICU admission
Readmission	Options: yes or no	Patient discharged from ICU and readmitted during the same hospital admission
Date of previous discharge†	yyyy/mm/dd	If readmission is yes; Previous ICU discharge date during same hospital admission
Type of admission *P**	Options: nonoperative, postoperative	Nonoperative - Admissions not arriving directly from OR or PACU including patients who may have undergone on operative procedure earlier in their hospital admission Postoperative - Admission directly from the OR or PACU
Emergency surgery *P* †	Options: yes or no	If type of admission is postoperative; Immediate surgery, where resuscitation (stabilization and physiological optimization) is simultaneous with surgical treatment and where surgery normally takes place within minutes of decision to operate or as deemed by surgical team
Reason for admission (operative) *P**	Type to search	If postoperative admission; Search for the operative procedure from the SNOMED CT1 list which most accurately describes what procedure was undertaken. If there have been multiple operative procedures, use the additional fields to add additional procedures
Reason for admission (disorder) *P* †	Type to search	If postoperative admission; In addition to the operative procedure, search and select any disorders from the SNOMED CT list which necessitated admission to the ICU.
Reason for admission (disorder) *P**	Type to search	If nonoperative; The main disorder which has necessitated admission to the ICU. This should be based only on what is known or suspected to be primary disorder within the first hour of admission. Any additional disorders deemed to be significant to the patient’s admission to the unit should be added using the additional reason for admission fields.
SARI diagnosis *S**	Options: confirmed. suspected, no	Indicate if at discharge the patient has a clinically suspected or laboratory confirmed severe acute respiratory infection as per WHO definition
If SARI confirmed or suspected - organism†	Dropdown list	If SARI is suspected or confirmed; Specify the organism identified as precisely as possible.
Type of test†	Dropdown list	If SARI confirmed; The type of test used to confirm the SARI diagnosis from a given list
Other type of test†	Text	If type of test is other; Any other type of test used to confirm the SARI diagnosis
Comorbidities *P**	Dropdown list	Conditions currently present or diagnosed prior to this hospital admission (CCI)
Comorbidities (Other)†	Type to search	If comorbidities is other; Search the SNOMED CT1 list and select any other conditions that are not available in the comorbidities dropdown list.
**Admission assessment**		
Screened for eligibility for MOTIVATE-ICU? *	Options: yes or no	Select whether the patient has been screened for inclusion in the study.
Age ≥ 15 years*	Options: yes or no	Record whether the patient is 15 years or older.
Transferred ≥ 24 hours after initiation of mechanical ventilation*	Options: yes or no	Record whether the patient was transferred in from non-study ICU after initiation of mechanical ventilation
Clinical frailty score (prior to critical illness)*	Dropdown list	*If enrolled in the MOTIVATE-ICU study* Number 1 - 9 indicating physical functionality and level of independence prior to current hospital admission or current illness 1. Very fit (regularly exercises) 2. Fit (occasionally exercises) 3. Managing well (not active beyond routine walking) 4. Vulnerable (independent but limited outside activities) 5. Mildly frail (needs help with heavy housework) 6. Moderately frail (needs help with bathing) 7. Severely frail (completely dependent but not high risk of dying) 8. Very severely frail (completely dependent, at end of life) 9. Terminally ill (life-expectancy<6months, not otherwise evidently frail)
Immunosuppressive treatment*	Options: yes or no	*If enrolled in the MOTIVATE-ICU study* Treatment known to cause immunosuppression; chemotherapy or steroids, AIDS
Height*	cm	*If enrolled in the MOTIVATE-ICU study* Measured height or length in cm (may be estimated)
Ventilation ***	Options: yes or no	Self-vent: No breaths are delivered by a mechanical device during the first hour of admission to ICU. Mechanical vent: All or some of the breaths or a portion of the breaths (pressure support) are delivered by a mechanical device during the first hour of admission to ICU.
Route of ventilation*	Dropdown list	*If* mechanical vent *; options are:* The type of mechanically assisted breathing during the first hour of admission. If multiple modes are used, please report the most invasive. ETT Tracheostomy NIV mask
Route of ventilation*	Dropdown list	*If self-vent; options are:* High flow oxygen (min flow 30/min) Tracheostomy Own airway
CPAP*	Options: yes or no	*If self-vent and enrolled in the MOTIVATE-ICU study* Record whether the patient received CPAP.
Primary Indication of IMV* ‡	Dropdown list	*If enrolled in the MOTIVATE-ICU study* Indications: upper airway protection, hypoxemic respiratory failure, hypercapnic respiratory failure, impending respiratory arrest, severe hemodynamic instability, cardiac arrest/post-cardiac arrest, post-anesthesia care, depressed level of consciousness
Mode of invasive ventilation* ‡	Dropdown list	PC-AC, VC-AC, PC-SIMV, VC-SIMV, PSV, PRVC, T-piece
Tidal volume* ‡	mL	*If enrolled in the MOTIVATE-ICU study* Set tidal volume if volume mode Measured expired tidal volume if pressure mode. Enter the tidal volume recorded in the 1st hour of initiation of ventilation. If not available or applicable, then the value closest to 08:00hrs.
Peak inspiratory pressure/inspiratory pressure* ‡	cmH2O	*If enrolled in the MOTIVATE-ICU study* Measured peak inspiratory pressure if volume mode Set inspiratory pressure if pressure mode Enter the pressure recorded in the 1st hour of initiation of ventilation. If not available or applicable, then the value closest to 08:00hrs.
FiO₂ *P**	/ or %	The highest recorded inspired oxygen concentration during the first hour of admission to ICU. If not available in the first hour, then the highest value in the first 24 hours should be recorded.
SpO₂ *P**	%	Percentage of oxygen-saturated hemoglobin during the first hour of admission to ICU. If not available in the first hour, the worst value in the first 24 hours should be recorded.
PaO₂ *P* †	mmHg or KPa	The partial pressure of oxygen measured in the arterial blood. The lowest recorded during the first hour of admission. If not available in the first hour, the lowest value in the first 24 hours should be recorded.
Arterial pH *P* †	pH	The measurement of the pH of plasma of an arterial blood sample. The most deranged reading during the first hour of admission must be recorded. This is the reading furthest from the normal range of pH 7.35 to 7.45. If not available, the worst in the first 24 hours.
Respiratory rate *P**	b/min	Self-vent on admission: first recorded RR on admission to the ICU. Mechanical vent on admission: last recorded RR prior to intubation.
Heart rate *P**	b/min	First recorded heart rate within the first hour of admission to the ICU
Systolic blood pressure *P**	mmHg	First recorded systolic BP within the first hour of admission to the ICU.
Diastolic blood pressure *P**	mmHg	First recorded diastolic BP within the first hour of admission to the ICU.
Temperature *P**	°F or °C	First recorded temperature within the first hour of admission to the ICU. If not available, the worst recorded value in the 1st 24 hours. Worst value being the farthest from the normal axillary range 36.0 - 37.5C or 96.8 - 99.5F
Sedated *P**	Options: yes or no	Use of sedative drugs for a minimum of one hour continuous infusion, or greater than one bolus.
Sedation medication*	Dropdown list	*If enrolled in the MOTIVATE-ICU study* Enter the sedatives used at initiation of ventilation Benzodiazepines Ketamine Propofol Dexmedetomidine/Clonidine Opioids
Sedation medication	Dropdown list	*If enrolled in the MOTIVATE-ICU study* Enter the sedatives used at initiation of ventilation Benzodiazepines Ketamine Propofol Dexmedetomidine/Clonidine Opioids
Sedation medication	Dropdown list	*If enrolled in the MOTIVATE-ICU study* Enter the sedatives used at initiation of ventilation Benzodiazepines Ketamine Propofol Dexmedetomidine/Clonidine Opioids
Glasgow coma scale (eye) *P**	Score 1 - 4	Self-vent on admission: the first recorded eye response for GCS score on admission to ICU. Mechanical vent on admission: The last recorded eye response for GCS immediately prior to intubation.
Glasgow coma scale (verbal)*	Score 1 - 5	Self-vent on admission: the first recorded verbal response for GCS score on admission to ICU. Mechanical vent on admission: the last recorded verbal response for GCS immediately prior to intubation.
Glasgow coma scale (motor)*	Score 1 - 6	Self-vent on admission: The first recorded motor response for GCS score on admission to ICU. Mechanical vent on admission: The last recorded motor response for GCS immediately prior to intubation.
Jaundice*	Options: yes or no	*If enrolled in the MOTIVATE-ICU study* Presence of conjunctival jaundice of any degree on admission or if not available, in the 1st 24 hours of admission.
**Antimicrobial use** *S**	Options: yes or no	Use of antimicrobial therapy. Even if only one dose has been administered this is considered as a ‘yes’.
Antimicrobial type†	Dropdown list	If Antimicrobial use is yes; The type of antimicrobial from a drug list.
Cardiovascular support *P**	Options: yes or no	Continuous intravenous; inotropic or vasoactive medication within the first hour of ICU admission.
Vasoactive therapy†	Dropdown list	If Cardiovascular support is yes; The type and dose of vasoactive drug used from the list of options.
**Renal replacement therapy** *P**	Options: yes or no	Use of renal replacement therapy. Any duration of RRT is considered as a ‘yes’. Either in the 1st hour of admission or in the first 24 hours of admission.
Urine output*	Options: < 200mL/day or 2, < 500mL/day, 3 ≥ 500mL/day	*If enrolled in the MOTIVATE-ICU study* Total urine output in the 1st 24 hours of ICU admission
Haemoglobin *P*	g/dL or g/L	Hb measured within the first hour of admission to ICU. If unavailable, provide the last reported Hb prior to admission (max 24 hours prior) or within first 24 hours of admission
Platelet *P* †	x10^9^/L or K/µL or 10^3/mm^3 or x10^3/μL or cells/μL or lakhs/mm^3^	Platelet count measured within the first hour of admission to ICU. If unavailable last reported platelet counts prior to admission (max 24 hours prior) or within first 24 hours of admission
Packed cell volume *P* †	% or /	Also called haematocrit. This is the volume percentage of red blood cells in blood measured within the first hour of admission to ICU. If unavailable, provide the last reported PCV prior to admission (max 24 hours prior) or within the first 24 hours of admission
Serum sodium *P* †	mEq/L or mmol/L	Blood sodium measured within the first hour of admission to ICU. If unavailable, provide the last reported blood sodium prior to admission (max 24 hours prior) or within the first 24 hours of admission
Serum potassium *P* †	mEq/L or mmol/L	Blood potassium measured within the first hour of admission to ICU. If unavailable, provide the last reported blood potassium prior to admission (max 24 hours prior) or within the first 24 hours of admission
Serum HCO3 *P* †	mEq/L or mmol/L	Blood bicarbonate measured within the first hour of admission to ICU. If unavailable, provide the last reported blood bicarbonate prior to admission (max 24 hours prior) or within the first 24 hours of admission
Serum creatinine *P* †	mg/dL or mmol/L or μmol/L or mg/L	Blood creatinine measured within the first hour of admission to ICU. If unavailable, provide the last reported blood creatinine prior to admission (max 24 hours prior) or within the first 24 hours of admission
Blood urea *P* †	mg/dL or mmol/L or g/L	Blood urea measured within the first hour of admission to ICU. If unavailable last reported blood urea prior to admission (max 24 hours prior) or within the first 24 hours of admission
White Blood cell count†	10^9/L or K/µL or cells/mm^3^or 10^3/mm^3^or other	WBC measured within the first hour of admission to ICU. If unavailable, last reported measurement prior to admission (max 24 hours prior) or within the first 24 hours of admission.
Total Bilirubin	umol/L or mg/dL	Total Serum bilirubin measured within the first hour of admission to ICU. If unavailable, last reported measurement prior to admission (max 24 hours prior) or within the first 24 hours of admission.
Chest X-ray/CT*	Options: yes or no	*If enrolled in the MOTIVATE-ICU study* Including chest CT-scan. Done during 1st 24 hours of ICU admission
Chest X-ray/CT findings*	Dropdown list	*If enrolled in the MOTIVATE-ICU study* *If ‘Chest X-ray’ or chest CT-Scan is ‘yes’:* *Normal, New Infiltrates, Persistent infiltrates, Consolidation, Cavitation, Pneumothorax, Pulmonary edema, Atelectasis, Pleural effusion, Pulmonary embolism*
**Discharge**		
Date of ICU discharge *P**	yyyy/mm/dd	The date on which the patient is discharged from or dies in the ICU.
Time of ICU discharge *P* †	hh:mm:ss	The time on which the patient is discharged from or dies in the ICU.
ICU discharge status *P**	Dropdown list	Whether the patient is alive at the point of ICU discharge.
Enrolled in MOTIVATE-ICU study?*	Options: yes or no	Record whether the patient has been enrolled in the MOTIVATE-ICU study
Family/ lay care provider in ICU	Options: yes or no	*If enrolled in the MOTIVATE-ICU study and consent was withdrawn* Record whether the patient had a family or family appointed lay care provider whilst in the ICU, who assisted with daily care activities
Consent withdrawn for MOTIVATE-ICU??*	Options: yes or no	*If enrolled in the MOTIVATE-ICU study and consent was withdrawn* Record whether the patient has been withdrawn from the MOTIVATE-ICU study.
Date withdrawn*	DateCor	*If enrolled in the MOTIVATE-ICU study and consent was withdrawn* Record the date the patient/legal representative withdrew consent. Consent must be withdrawn within 72 hours of ICU admission.
Time withdrawn*	Time	*If enrolled in the MOTIVATE-ICU study and consent was withdrawn* Record the time the patient/legal representative withdrew consent. Consent must be withdrawn within 72 hours of ICU admission.
Primary Indication for tracheostomy*	Dropdown list	*If enrolled in the MOTIVATE-ICU study and consent was not withdrawn* Weaning failure Anticipated prolonged MV Prolonged MV Upper airway obstruction Airway protection Not known. No tracheostomy performed
Tracheostomy type*	Dropdown list	*If enrolled in the MOTIVATE-ICU study and consent was not withdrawn and ‘primary indication for tracheostomy’ is not ‘no tracheostomy performed’* surgical trach Percutaneous trach
Tracheostomy complications*	Dropdown list	*If enrolled in the MOTIVATE-ICU study and consent was not withdrawn* SSI Major bleeding Dislodgement Malfunction None
Decannulation date	yyyy/mm/dd	*If enrolled in the MOTIVATE-ICU study and consent was not withdrawn* Date the tracheostomy was decannulated.
End-of-life care and/or ICU palliative support*	Options: yes or no	*If enrolled in the MOTIVATE-ICU study and consent was not withdrawn* Record whether the patient was admitted for end-of-life care and/or ICU palliative support or determined to be for end-of-life care
Date care became palliative*	Date	*If ‘end-of-life care and/or ICU palliative support’ is ‘yes’* Record the date the patient was determined to be for end-of-life care and/or ICU palliative support.

* failure to save alert; † pre saving alert; ‡ If on invasive mechanical ventilation.

Admission

P = used to calculate standardized mortality ratio. Captured close to time admission, calculated using E TropICS or APACHE II.

ID = used to identify encounters during the inpatient episode. Replaced with a unique platform encounter ID once encounter completes. S = used for infection surveillance.

References:

1. Donnelly K. SNOMED-CT: The advanced terminology and coding system for eHealth. Stud Health Technol Inform. 2006;121:279-90.

2. ICNARC Case Mix Programme Data Collection Manual v3.1.

3. Ho KM, Dobb GJ, Knuiman M, Finn J, Lee KY, Webb S. A comparison of admission and worst 24-hour Acute Physiology and Chronic Health Evaluation II scores in predicting hospital mortality: a retrospective cohort study. Crit Care. 2006;10(1):R4.

**Table 2SB:** Daily variables

Daily Q Assessment Completed every day for the inpatient ICU stay up to 28 days. Data reflects events for the previous 24 hours: 00:00 - 24:00hrs.		
Care of the ventilated patients		
**Variable name**	**Data entry format**	**Definition for collection**
Enrolled in MOTIVATE-ICU study?*	Options: yes or no	Record whether the patient has been enrolled in the MOTIVATE-ICU study. If the patient was not eligible, or the patient/representative withdrew consent within 72 hours of admission, please select ‘no’.
Ventilation*	Options: yes or no	Self-vent: no breathing is delivered by a mechanical device for a period of one hour during the day. Mechanical vent: all or some of the breaths or a portion of the breaths (pressure support) are delivered by a mechanical device for one hour during the day. Please record the highest ventilation support in the last 24 hours
Route of ventilation*	Dropdown list	*If mechanical vent; options are:* The type of mechanically assisted breathing during the first hour of admission. If multiple modes are used, please report the most invasive. ETT Tracheostomy NIV mask
Route of ventilation*	Dropdown list	*If* self-vent; *options are:* High flow oxygen (min flow 30/min) Tracheostomy Own airway
CPAP*	Options: yes or no	*If self-vent and enrolled in the MOTIVATE-ICU study* Record whether the patient received CPAP.
Primary Indication of IMV* ‡	Dropdown list	*If enrolled in the MOTIVATE-ICU study* Indications: upper airway protection, hypoxemic respiratory failure, hypercapnic respiratory failure, impending respiratory arrest, severe hemodynamic instability, cardiac arrest/post-cardiac arrest, post-anesthesia care, depressed level of consciousness
Mode of invasive ventilation* ‡	Dropdown list	PC-AC, VC-AC, PC-SIMV, VC-SIMV, PSV, PRVC, T-piece
Tidal volume* ‡	ml	*If enrolled in the MOTIVATE-ICU study* Set tidal volume if volume mode Measured expired tidal volume if pressure mode. The tidal volume closest to 10am should be recorded.
Peak inspiratory pressure/ inspiratory pressure* ‡	cmH2O	*If enrolled in the MOTIVATE-ICU study* Measured peak inspiratory pressure if volume mode Set inspiratory pressure if pressure mode The peak inspiratory pressure/inspiratory pressure closest to 10am should be recorded.
Tracheal secretions*	Dropdown list	*If enrolled in the MOTIVATE-ICU study* *If ‘mechanical vent’ is yes;* *Appearance of ETT/TT secretions* *− Purulent* *− Non-purulent* *− Minimal/none*
Spontaneous breathing trial (SBT)	Options: yes or no	*If ‘mechanical vent’ is yes: and* *‘Route of ventilation’ is ‘tracheostomy’: or ‘ETT’:* A trial of spontaneous breathing, defined as one of the following four; − Trial of ‘T’ piece − Pressure support with low support (PS of 5 - 8) − CPAP (i.e. only PEEP between 0 - 5) − Automatic tube compensation with CPAP − Not eligible for SBT
Lowest FiO_2_	% or ratio ≤ 1.0	*If ‘mechanical vent’ is yes:* Lowest inspired oxygen concentration delivered for a minimum of one hour for the last 24 hours
Lowest PEEP	mmH_2_O	*If ‘mechanical vent’ is yes:* Lowest positive end expiratory pressure for a minimum of one hour for the last 24 hours
SpO_2_	%	The lowest peripheral oxygen saturation measured by pulse oximetry in the last 24 hours.
Respiratory rate highest	b/min	The highest measured respiratory rate recorded in the last 24 hours. Either set RR if no spontaneous breathing or total respiratory rate (set RR + spontaneous RR)
VTE prophylaxis	Dropdown list	*If ‘mechanical vent’ is yes:* Use of mechanical or pharmacological prophylaxis in the prevention of venous thromboembolism.
Contraindication	Dropdown list	*If ‘VTE prophylaxis’ is ‘none’:* The contraindication preventing the use of mechanical and pharmacological prophylaxis.
Stress ulcer prophylaxis	Options: yes or no	*If ‘ventilation’ is ‘mechanical vent’:* A drug is prescribed for the purpose of stress ulcer prophylaxis
Drug	Dropdown list	*If ‘stress ulcer prophylaxis’ is ‘yes’:* The named drug from the provided list used for stress ulcer prophylaxis.
*Inadvertent/Accidental extubation**	*Options: yes or no*	*If ‘mechanical vent’ is yes:* *The unexpected or unplanned extubation of a patient requiring reintubation* .
Suspected or confirmed vent tube blockage*	Options: 1. yes, and tube replaced 2. yes, and patient extubated/ decannulated, 3. no	*If ‘mechanical vent’ is yes:*
Head of bed >30° or patient sat up (if no degree measure)*	Options: yes or no	*If ‘mechanical vent’ is yes:* *Position of the head of bed* Please record head of bed position daily based on observed elevation at time of review. If no degree measures, patients have hip flexion with chest and head elevated using pillows or have total bed tilt (Trendelenburg).
Chest X-ray*	Options: yes or no	Chest X-ray done in the last 24 hours
Chest CT*	Options: yes or no	*If enrolled in the MOTIVATE-ICU study* Chest CT done in the last 24 hours
Chest X-ray/CT findings*	Dropdown list	*If enrolled in the MOTIVATE-ICU study* *If ‘chest X-ray’ or chest CT-scan is ‘yes’:* *Normal, new infiltrates, persistent infiltrates, consolidation, cavitation, pneumothorax, pulmonary edema, atelectasis, pleural effusion, pulmonary embolism*
Pulmonary complications*	Dropdown list	*None, new infiltrates, persistent infiltrates, pneumothorax, pleural effusion, atelectasis , development of ARDS, cardiogenic pulmonary edema, pulmonary infection*
**CVS care**		
Highest temperature	°F or °C	Highest recorded temperature
Cardiovascular support	Options: yes or no	Use of continuous intravenous inotropic and/or vasoactive medication for a minimum period of one hour.
Vasoactive drugs increased	Options: yes or no	*If ‘cardiovascular support’ is ‘yes’:* An increase in the use of vasoactive medication (either number of agents or dosage of an agent) for a minimum period of one hour.
Indwelling devices	Options: new, *insitu* or no	The presence of an indwelling catheter Catheter types − Central venous catheter − Hemodialysis catheter − Arterial catheter − Peripheral cannula − Urinary catheter − Chest drain − Abdominal (or other drain).
Site	Dropdown list	*If ‘central venous catheter’, ‘arterial catheter’ or ‘peripheral cannula’ or ‘urinary catheter’ is ‘new’ or ‘in situ’:* Select the location of the new or existing central venous catheter, arterial catheter or peripheral cannula.
Daily cumulative fluid balance*	Numerical (-10000 to +10000mL)	*If enrolled in the MOTIVATE-ICU study* Recorded from the patient fluid monitoring chart record from 1st 24 hours
**Renal care**		
**Renal replacement therapy**	Options: yes or no	Use of renal replacement therapy. Any duration of renal replacement therapy is considered as ‘yes’.
Type of the therapy	Dropdown list	*If ‘renal replacement therapy’ is ‘yes’:* The type of renal replacement therapy used.
**Neuro care**		
**Sedated**	Options: yes or no	Use of sedative drugs for a minimum of one hour. If bolus sedation, then 2 or more bolus.
Sedation medication*	Dropdown list	*If enrolled in the MOTIVATE-ICU study & yes to sedated* Enter the sedatives used at initiation of ventilation Benzodiazepines Ketamine Propofol Dexmedetomidine/Clonidine Opioids
Sedation medication	Dropdown list	*If enrolled in the MOTIVATE-ICU study & yes to sedated* Enter the sedatives used at initiation of ventilation Benzodiazepines Ketamine Propofol Dexmedetomidine/clonidine Opioids
Sedation medication	Dropdown list	*If enrolled in the MOTIVATE-ICU study & yes to sedated* Enter the sedatives used at initiation of ventilation Benzodiazepines Ketamine Propofol Dexmedetomidine/Clonidine Opioids
Sedation medication	Dropdown list	*If enrolled in the MOTIVATE-ICU study & yes to sedated* Enter the sedatives used at initiation of ventilation Benzodiazepines Ketamine Propofol Dexmedetomidine/Clonidine Opioids
Spontaneous awakening trial or (sedation hold).	Options: yes or no	*If ‘sedated’ is ‘yes’:* Spontaneous awakening trial (SAT or “sedation hold”) a period during which sedating medications that are being used to treat the patient were held in order to determine whether the patient requires ongoing sedation or can be managed without sedatives.
**Target RASS**	Dropdown list	*If ‘mechanical vent’ is yes and ‘sedated’ = yes* The target RASS is a measure of sedation, awakeness and agitation. The target RASS is the target set each day on the ward round. The options are available as a drop down. *+4 to -5 and ‘no target set’*
**Actual RASS score**	Dropdown list	*If ‘ventilation’ is ‘mechanical vent’ and ‘sedated’ = yes* RASS is a measure of sedation, awakeness and agitation. The actual RASS is the measured (observed) `RASS either immediately prior to a SAT or between 0800 -1200 hours. The options are available as a drop down. *+4 to -5 and ‘not recorded’*
**Skin care**		
**Pressure injury**	Options: yes or no	Pressure/ compression related injury to skin
Site of pressure injury	Dropdown list	*If ‘pressure injury’ is ‘yes’:* − Back of the head − Ears, face, mouth − Shoulders − Elbows − Lower back − Buttocks/ sacrum − Hips − Inner knees − Heels − Other
Grade	Dropdown list	*If ‘pressure injury’ is ‘yes’:* 1, 2, 3, 4, 5
**Infection management**		
**Highest white cell count**	x10^9^/L or x10^3^/mm^3^or x10^3^/μL	The highest recorded serum white blood cell count.
**Lowest white cell count**	x10^9^/L or x10^3^/mm^3^or x10^3^/μL	The lowest recorded serum white blood cell count.
**Infection**	Dropdown list	Presence of a confirmed or suspected infection.
Source	Dropdown list	*If ‘infection’ is ‘suspected’ or ‘confirmed’:* The source of infection from a list of body systems.
Clinician treating as healthcare associated infection	Options: yes or no	Clinicians may be treating patients for healthcare associated infection with or without microbiological confirmation. Information used to help interpret abx choice and perceived incidence of HCAI.
**Antimicrobial use daily**	Options: yes or no	Use of antimicrobial therapy. Even if only one dose has been administered, this is ‘yes’ Denominator- all ICU patients Eligibility = all.
Antimicrobial type	Dropdown list	*If ‘Antimicrobial use daily’ is ‘yes’:* The type of antimicrobial from a drug list.
**Microbiology**		
**Culture obtained today**	Options: yes or no	Was a culture sample taken today.
Type of culture	Dropdown list	Type of culture. *1, Blood | 2, Urine | 3, CSF | 4, Stool | 5, Sputum | 6, BAL | 7, Wound*
**Culture report**	Dropdown list	*1, Growth | 2, No growth | 3, Pending | 4, None Obtained | 5, None Pending* Record whether a new culture report was available today. Growth: if a new culture report was available today and growth was identified. No growth: if a new culture report was available today and no growth was identified. Pending: if a culture report was obtained and results are pending None obtained: if no cultures have been obtained during the patient’s say None pending: if results of all cultures taken are available and have been previously entered on the patient’s record.
Date culture taken	yyyy/mm/dd	*If ‘culture report’ is ‘growth’ or ‘no growth’:* Record the date the specimen was taken.
Organism	Dropdown list	*If ‘culture report’ is ‘growth’:* Organism list referred by HELICS (Note: refer Organism list sheet)
Resistance name	Dropdown list	*If ‘Organism’ is recorded:* From the dropdown list, record the antimicrobial resistance for the organism
Sensitivity name	Dropdown list	*If ‘Organism’ is recorded:* From the dropdown list, record the antimicrobial sensitivity for the organism
**Mobilization and rehabilitation**		
Physiotherapy/Mobilisation	Dropdown list	*If ‘mechanical vent’ is yes* ’: Patients ventilated may receive passive or active physiotherapy in the ICU. The type of physiotherapy provided should be recorded daily. The event may occur at any time in the previous 24 hours. Some patients may not be eligible for daily physio (for example due to cardiovascular instability, or because they are at the end of life or because they are receiving a procedure). *1. On bed physiotherapy* *2. Out of bed mobilisation* *3. No physiotherapy delivered* *4. No physiotherapy delivered due to patient not eligible*

* failure to save alert; ‡ If on invasive MV.

References:

1. Donnelly K. SNOMED-CT: The advanced terminology and coding system for eHealth. Stud Health Technol Inform. 2006;121:279-90.

2. ICNARC Case Mix Programme Data Collection Manual v3.1.

3. Ho KM, Dobb GJ, Knuiman M, Finn J, Lee KY, Webb S. A comparison of admission and worst 24-hour Acute Physiology and Chronic Health Evaluation II scores in predicting hospital mortality: a retrospective cohort study. Crit Care. 2006;10(1):R4.

**Table 3S:** Modified SOFA score

SOFA score	0	1	2	3	4
Respiratory*					
PaO_2_/FiO_2_	> 400	300 - 400	200 - 299	100 - 199	≤ 100
SpO_2_/FiO_2_		316 - 400	236 - 315	151 - 235	≤ 150
Coagulation (platelets 10^3^/mm^3^)	> 150	< 150	< 100	< 50	< 20
Liver					
Bilirubin (mg/dL)	< 1.2	1.2 - 1.9	2.0 - 5.9	6.0 - 11.9	> 12.0
Scleral icterus/Jaundice*	No			Yes	
Cardiovascular					
Hypotension (mcg/kg/min)	No hypotension	MAP < 70	Dobutamine (any dose)	Norepinephrine/ epinephrine ≤ 0.1	Norepinephrine/ epinephrine > 0.1
CNS					
Glasgow coma score	15	13 -14	10 - 12	6 - 9	< 6
Renal					
Creatinine (mg/dL)	< 1.2	1.2 - 1.9	2.0 - 3.4	3.5 - 4.9	> 5.0
Urine output (mL/d)				< 500	< 200

SOFA - Sequential Organ Function Assessment score; PaO_2_- partial pressures of oxygen in plasma; SpO_2_- peripheral saturation of oxygen; FiO_2_- fraction of inspired oxygen; CNS - central nervous system. * Modified to include SpO_2_/FiO_2_ratio, clinical jaundice for the liver dysfunction if no blood gas analysis or Liver function tests.

Reference: Rahmatinejad Z, Reihani H, Tohidinezhad F, Rahmatinejad F, Peyravi S, Pourmand A, et al. Predictive performance of the SOFA and mSOFA scoring systems for predicting in-hospital mortality in the emergency department. Am J Emerg Med. 2019;37(7):1237-41.

**Table 4S:** Electronic Case Report Form for intensive care unit organizational characteristics for Redcap

Baseline information	
1. Hospital name	Open text
2. Location (dropdown)	a. Kampala b. Gulu c. Mbarara d. Mbale e. Jinja
3. Designation of the person providing the information (dropdown menu)	a. Consultant ICU b. Fellow ICU c. Resident ICU d. Medical officer e. Nursing in-charge f. Nurse, Technician
4. Contact e-mail	Open text
**Hospital characteristics Please, provide the following information that describes your hospital MOST appropriately:**	
**Variable**	**Data to be collected**
1. Type of hospital (dropdown menu)	Public PNFP Private
1.1 University-affiliated?	Yes/No
2. Number of hospital beds (i.e., active hospital beds) (estimate if not certain)	Open text
3. Total number of intensive care units in the hospital (including cardiac and coronary care, but excluding intermediate, and step-down units) Intensive care unit definition: intensive care unit as a designated unit within a hospital that routinely provides invasive mechanical ventilation therapy with continuous vital sign monitoring (electrocardiographic monitoring, heart/pulse rate, noninvasive blood pressure, peripheral oxygen saturation) and designated nursing care for each bed in the unit i.e, at least three patients per week for at least 24 hours.	Open text
4. Is (are) there intermediate or step-down unit(s)?	Yes/No
5. Is there an Emergency Room or Department? (dropdown)	No Yes, open Yes, referenced
6. Is the hospital certified by an Accreditation Organization? (dropdown)	No Yes, national Yes, international
7. Is there a hospital wide rapid response team formally implemented for ≥ 6 months? (dropdown)	Yes/No
8. Are there training programs or medical residence at your institution? (dropdown)	No Yes, but not in critical care Yes, including critical care
9. Are there training programs for graduate nurses at your institution? (dropdown)	No Yes, but not specific to critical care Yes, including specific training in critical care
10. Are there training programs for other care providers (physiotherapists, psychologists, clinical pharmacists or nutritionists) at your institution?	No Yes, but not specific to critical care Yes, including specific training in critical care
**Intensive care unit characterization form Please, provide the following information that describes your intensive care unit MOST appropriately. If there is more than one intensive care unit at your hospital, please, fill an “intensive care unit form” for each of them**	
Intensive care unit name:	Open text
**I - ICU characterization**	
1. ICU type (dropdown)	General or mixed medico-surgical; surgical; medical; neurological; coronary or cardiac; other
2. How long has your ICU been functional?	Open text
3. Number of active ICU beds *(Beds used for continuous monitoring, mechanical ventilation and other organ support services)*	Open text
4. Estimated number of ICU admissions in past one year	Open text
5. Organ support capacity in addition to invasive mechanical ventilation within the ICU (checklist)	− Pharmacological cardiovascular support (inotropes and vasopressors) − IABP − ECMO − Blood gas analysis − Intermittent renal replacement therapy − Continuous renal replacement therapy − Parenteral nutrition
6. Organ monitoring capacity in addition to noninvasive monitoring within the ICU (checklist)	− CVP − ABP − Invasive cardiac output monitoring − Pulmonary artery catheterization − Invasive and/or noninvasive cerebral monitoring (NIRS, ICP monitoring, EEG) − In-house ultrasonography monitoring
**II - Staffing patterns: clinicians**	
7. ICU model type *Open unit: primary doctors make all care-related decisions in the ICU with the ICU doctor on consult as needed.* *Closed unit: ICU doctor makes all care-related decisions.* *Semi-closed: shared decision making between ICU doctor and primary doctor*	− Open unit − Closed unit − Semi-closed
8. Primary speciality of the consultant in charge of the ICU? (dropdown)	− Anesthesiology, Internal Medicine, Emergency medicine, Surgery, Pulmonology, Cardiology, Nephrology, Non-specialized
9. Is the Consultant in charge of ICU an intensivist (trained & certified in ICM for at least one year)?	− Yes/No
9.1 If no, is the consultant in charge of ICU a non-intensivist physician but with ≥ 2 years of ICU clinical experience?	− Yes/No
10. Which of the following specialists are available onsite in the hospital for ICU consultation? (checklist)	− Anesthesiologist, Cardiologist, Nephrologist, General surgeon, General physician, Pulmonologist, Infectious disease, Microbiologist, Obstetrician-gynecologist, Gastroenterologist, Neurologist, Hematologist, Pediatrician
11. Total number of ICU doctors in your ICU	− Open text
12. Qualification of ICU doctors employed in the ICU? (yick all that apply)	− Anesthesiology, Internal Medicine, Emergency medicine, Surgery, Pulmonology, Cardiology, Nephrology, Non-specialized, Specialist trainees (Specify - Anesthesia, EM, IM, Surgery), Medical officers
13. Do you have staff Intensivists in your ICU? (Intensivist = Base specialty in IM, Anesthesia, EM, Surgery with ≥1-year certified training in Intensive care medicine)	− No/Yes
13.1 If yes, what is the most frequent coverage pattern on weekdays?	1. 24/7 in-house 2. Daytime with nighttime on-call 3. Daytime with nighttime anesthesiologist 4. Daytime with nighttime non-anesthesia specialist 5. Daytime with nighttime trainee 6. Daytime with nighttime medical officer 7. Daytime with nighttime AHP 8. Tele-ICU
13.2 If yes, what is the most frequent coverage pattern on weekends?	1. 24/7 in-house 2. Daytime with nighttime on-call 3. Daytime with nighttime anesthesiologist 4. Daytime with nighttime non-anesthesia specialist 5. Daytime with nighttime trainee 6. Daytime with nighttime medical officer 7. Daytime with nighttime AHP 8. Tele-ICU 9. None
14. Do you have staff non-intensivist specialty physicians in your ICU with at least 2 years’ ICU clinical experience?	9. No/Yes
14.1 If yes, what is the most frequent coverage pattern on weekdays?	1. 24/7 in-house 2. Daytime with nighttime anesthesiologist 3. Daytime with nighttime non-anesthesia specialist 4. Daytime with nighttime trainee 5. Daytime with nighttime medical officer 6. Daytime with nighttime AHP 7. Tele-ICU
14.2 If yes, what is the most frequent coverage pattern on weekends?	1. 24/7 in-house 2. Daytime with nighttime anesthesiologist 3. Daytime with nighttime non-anesthesia specialist 4. Daytime with nighttime trainee 5. Daytime with nighttime medical officer 6. Daytime with nighttime AHP 7. Tele-ICU 8. None
**Staffing patterns: nurses**	
15. Is the ICU In-charge nurse certified in Intensive Care nursing from an accredited program?	Yes/No
15.1. If the incharge nurse isn’t certified in Intensive care nursing; does he/she have ≥ 2 years of ICU clinical experience?	Yes/No
16. Total Number of nurses working in the ICU	Open text
17. Number of nurses certified in Intensive care nursing	Open text
18. Number of nurses (not certified) with ≥ 2 years of ICU clinical experience	Open text
19. Maximum number of self-ventilated patients 1 nurse looks after during the day (n)	Open text
20. Maximum number of ventilated patients 1 nurse looks after during the day (n)	Open text
21. Maximum number of self-ventilated patients 1 nurse looks after during the night (n)	Open text
22. Maximum number of ventilated patients 1 nurse looks after during the night (n)	Open text
23. Maximum number of self-ventilated patients 1 nurse looks after during the weekend (n)	Open text
24. Maximum number of ventilated patients 1 nurse looks after during the weekend (n)	Open text
25. Are there critical care trained nurses present in the ICU during night shift?	Yes/No
26. Are there critical care trained nurses present in the ICU during weekends?	Yes/No
**Staffing patterns: physiotherapists and respiratory therapists (* excluding trainees and residents)**	
27. Does the ICU team have access to physiotherapists?	No; yes, but not dedicated to the ICU; yes, dedicated to the ICU
27.1 In case of dedicated physiotherapists, they are present in the ICU	Only during day shifts; during day and night shifts
**Staffing patterns: other care providers (* excluding trainees and residents)**	
28. Nutritionist	No; yes, but not dedicated to the ICU; yes, dedicated to the ICU
29. Clinical pharmacist	No; yes, but not dedicated to the ICU; yes, dedicated to the ICU
**III - Empowerment assessment of the multidisciplinary team**	
Are your ICU healthcare providers allowed to proceed with the following activities WITHOUT THE NEED of direct discussion with the intensive care physician, except in exceptional cases: **Check:** No: the healthcare provider CANNOT proceed with the activity without explicit communication to the ICU physician. Sometimes: the healthcare provider CAN SOMETIMES proceed with the activity without explicit communication to the ICU physician in certain occasions or patients. Yes: the healthcare provider CAN ALWAYS proceed with the activity without explicit communication to the ICU physician and is at his/her discretion to judge if the intensivist must be communicated in advance.	
**Nurses (*except residents and postgraduate students)**	
30. Pause or titrate sedation, according to routine or protocol 31. Increase diet infusion, according to routine or protocol 32. Titrate vasopressors, directed to or based on goals 33. Start ventilatory weaning, according to routine/protocol 34. Increase FiO_2_(by either invasive or noninvasive device) 35. Start out of be active mobilization, according to routine/ protocol 36. Offer symptomatic prescribed as “if necessary” (SOS) 37. Provide patient information to the family/next of kin	No; sometimes; yes
**Physiotherapists (*except residents and postgraduate students)**	
38. Start ventilatory weaning, according to routine/protocol 39. Modify ventilator parameters and FiO_2_, including PEEP 40. Increase or decrease oxygen support, including changing noninvasive devices (e.g. changing nasal catheter to Venturi mask) 41. Start noninvasive ventilation or high-flow oxygen nasal cannula 42. Perform alveolar recruitment maneuver 43. Start routine passive mobilization 44. Start active mobilization (including out of bed), according to routine/ protocol, including in ventilated patients	No; sometimes; yes
**IV - Organizational aspects and patient care processes**	
**Multidisciplinary rounds**	
45. Do the rounds occur at the bedside?	No; sometimes; yes, always
46. Are multidisciplinary clinical rounds performed?	No Yes, on some days of the week; Yes, every day except weekends; Yes, every day, including weekends
46.1 If yes, which professionals participate in multidisciplinary rounds? (check all that apply)	Physicians, nurses, physiotherapists, Psychologists, clinical pharmacist, nutritionists
**Checklists**	
47. Are checklists used during clinical rounds to assist with patient care and management?	No Yes, on some days of the week; Yes, daily, except weekends; Yes, daily, including weekends
47.1. If yes, what are the functions of checklists in clinical rounds?	Only monitor adherence to best practices; Monitor and guide the implementation of adherence to best practices
**Continuity assurance procedures of patient care information during handover**	
48. What is the handover model in your ICU?	Individual, each team of health professionals do the handover separately; Multidisciplinary, all health professionals in the ICU do the handover together
49. Is there STRUCTURED formal documentation of the handover in written record?	Yes/No
49.1 If yes, what avenue is used? (check all that apply)	Form or template on paper Electronic media
49.1.1 If yes, specify	Open text
**V - Organizational aspects and patient care processes**	
**Clinical protocols**	
In the last 6 months, clinical care protocols are being implemented in your ICU? **Protocol** is defined as an explicit, standardized and detailed written plan or clinical pathway that provides a set of guiding rules for caring for patients with a given condition. **Check:** No: no protocol OR protocol not implemented Partially implemented: the protocol was elaborated, but the teams have not been adequately trained and there is no regular monitoring of indicators. Fully implemented: the protocol has been elaborated and is fully implemented, with adequate structure, processes, actions, strategies, team training, as well as the regular monitoring and reporting of the indicators.	
50. Sedation (i.e., daily interruption of sedation or protocol- directed sedation in ventilated patients)	No; partially; fully
51. Liberation from mechanical ventilation (i.e., health care-driven spontaneous breathing trials)	No; partially; fully
52. Protective ventilation (i.e., ventilation with low tidal volumes in patients with acute lung injury or ARDS)	No; partially; fully
53. Fluid management protocols (i.e., implementation of conservative fluid management protocols including diuresis)	No; partially; fully
54. Prevention of catheter-related bloodstream infection (i.e., implementation of checklists during insertion and maintenance of intravascular catheters)	No; partially; fully
55. Prevention of ventilator-associated pneumonia VAP (i.e., implementation of best practices for the prevention of VAP in ventilated patients)	No; partially; fully
56. Early mobilization of ventilated patients (i.e., protocoled early mobilization and exercise (including out-of- bed exercises), including physical and occupational therapy during periods of sedation interruption in ventilated patients)	No; partially; fully
57. Hand-washing protocol (i.e,, soap and water as well as alcohol-hand rub during patient care)	No; partially; fully

**Table 5SA:** Intensive care unit classification scoring matrix

	Level 1 score	Level 2 score	Level 3 score	Weight
**Criteria**				
ICU model-type	Open	Semi-closed	Closed	2
ICU director	0 (specialist^a^< 2 years OR non-specialist)	1 (specialist ≥ 2 years ICU clinical experience)	2 (certified intensivist)	3
Staffing: physician coverage				
24/7	0 (specialist < 2 years OR non-specialist)	1 (specialist ≥ 2 years ICU clinical experience)	2 (certified intensivist)	3
Day time	0 (specialist < 2 years OR non-specialist)	1 (specialist ≥ 2 years ICU clinical experience)	2 (certified intensivist)	3
Night time	0 (MO, AHP, on-call)	1 (non-anesthesia specialist, specialist trainee)	2 (anesthesiologist)	2
Staffing: medical specialists	0 (< 25% on-site)	1 (25 - 50% on-site	2 (> 50% on-site)	2
Staffing: nursing coverage				
ICU in-charge nurse	0 (< 2 years ICU clinical experience)	1 (≥ 2 years ICU clinical experience)	2 (certified ICU training)	3
Staffing: nursing experience	0 (< 50% nurses with ≥ 2 years ICU clinical experience)	1 (≥ 50% nurses with ≥ 2 years ICU experience)	2 (≥ 50% nurses with certified ICU training)	3
Staffing: patient-to-nurse ratio	0 (≥ 3:1)	1 (≤ 2:1)	2 (1:1)	3
Allied health personnel				
Physiotherapist	0 (not available)	1 (available, not dedicated)	2 (dedicated to ICU)	2
Nutritionist	0 (not available)	1 (available, not dedicated)	2 (dedicated to ICU)	2
Clinical pharmacist	0 (not available)	1 (available, not dedicated)	2 (dedicated to ICU)	2
Monitoring				
Basic	0 (noninvasive)	1 (level 1 + invasive BP, CVP + blood gas analysis)	2 (level 2 + cardiac output, cerebral monitoring, in-house ultrasound)	3
Organ support: ventilation	0 (no IMV support, only non-invasive O_2_therapy)	1 (level 1 + intermittent RRT OR vasoactive support OR TPN)	2 (level 2 + ECMO OR CRRT OR IABP)	3
Rounds				
MDT	No MDT round	Only physicians + nurses < 5 days/week	Physician & nurses + physiotherapists OR nutritionist OR clinical pharmacist ≥ 5 days/week	3
Integration with ICU outreach				
Rapid Response Team	0 (none)		Formal RRT present	2
HDU/stepdown unit	0 (none)		Present	2
Quality improvement				
Clinical protocol implementation	0 ( *ad hoc* OR no protocols OR no implemented protocols OR < 50% partial implementation OR < 25% full implementation)	1 (50 - 75% partial implementation OR 25 - 50% full implementation)	2 (full implementation of > 50% OR > 75% partial implementation)	3

ICU - intensive care unit; MO - medical officer; AHP - allied health personnel; BP - blood pressure; CVP - central venous pressure; IMV - invasive mechanical ventilation; RRT - renal replacement therapy; TPN - total parenteral nutrition; ECMO - extracorporeal membrane oxygenation; CRRT - continuous renal replacement therapy; IABP - intraaortic balloon pump; RRT - Rapid Response Team; HDU - high dependency unit.^a^Specialist - graduate from a formal specialist training training program after first medical degree

Source: Marshall JC, Bosco L, Adhikari NK, Connolly B, Diaz JV, Dorman T, et al. What is an intensive care unit? A report of the task force of the World Federation of Societies of Intensive and Critical Care Medicine. J Crit Care. 2017;37:270-6 **.** ^(36)^

**Table 5SB:** Guide - steps to apply the scoring matrix

1. Assign scores:
a. For each criterion, assign scores (0, 1, or 2) based on the survey data for each ICU
2. Calculate weighted scores:
a. Multiply the scores by the corresponding weights
3. Sum total scores:
a. Add all weighted scores to obtain the total score for each ICU.
4. Determine ICU level:
a. Categorize each ICU based on thresholds:
i. Level 1: ≤ 33% of the maximum score
ii. Level 2: 34 - 66% of the maximum score
iii. Level 3: ≥ 67% of the maximum score

ICU - intensive care unit.
