# Supplementary material for: Potentially MOdifiable factors To ImproVe outcomes of mechanically Ventilated patients in a low-income country Intensive Care Units (MOTIVATE-ICU): rationale and protocol for a registry-embedded prospective observational study
Source: Crit Care Sci. 2025 Sep 22;37:e20250273. doi: 10.62675/2965-2774.20250273 (PMC12614988; doi:10.62675/2965-2774.20250273)
Supplement: Supplementary file 1 [file 2965-2774-ccsci-37-e20250273-suppl01.pdf]

Critical Care Science
https://doi.org/10.62675/2965-2774.20250273

Potentially MOdifiable factors To ImproVe outcomes of mechanically Ventilated patients in a low-income country Intensive Care Units (MOTIVATE-ICU): rationale and protocol for a registry-embedded prospective observational study

Cornelius Sendagire[1,2,3,4], Luigi Pisani[5], Alice Nuwagira[3], Adam Hewitt-Smith[6,7], Jane Nakibuuka[8], Herbert Kiwalya[7], Nodreen Christine Ayupo[4], Dominic Ogwal[9], Dennis Kakaire[10], Patience Atumanya[11,12], Betty Khainza[13], Hajara Nakayiza[14], Hawa Nakandi[15], Kenneth Tomanya[16], Martha Alupo[8], Lameck Ssemogerere[17], Erasmus Okello[18], Aggrey Lubikire[19], Andrew Kintu[20], Innocent Nyeko[21], Andrew Kamau[22], Chamira Kodippily[5], Arthur Kwizera[1], Abigail Beane[5], Rashan Haniffa[5], Jorge Ibrain Figueira Salluh[2]

Table 1S - List of current intensive care units in Uganda - 2023

Hospital	ICU type	Location	#ICU beds	#Admissions/month
Mulago NRH	GICU	Kampala	27	20
Mbarara RRH	GICU	Mbarara	8	12
Lubaga Hospital	GICU	Kampala	4	10
Mbale Regional Referral Hospital	GICU	Mbale	5	5
Kiruddu NRH	MICU	Kampala	4	8
Nakasero Hospital	GICU	Kampala	8	15
Uganda Heart Institute	CVICU	Kampala	4	8
Kampala Hospital	GICU	Kampala	6	8
Nsambya Hospital	GICU	Kampala	5	8
Case Hospital	GICU	Kampala	9	8
Medipal International Hospital	GICU	Kampala	10	5
Medical Hub	GICU	Kampala	4	4
TMR International Hospital	GICU	Kampala	9	10
Cure Hospital Kampala	Neuro-ICU	Mbale	5	8
Lacor Hospital	GICU	Gulu	8	12
UMC Victoria Hospital	GICU	Kampala	5	8
Platinum Hospital	GICU	Kampala	4	6
International Hospital Kampala	GICU	Kampala	11	12
Kawempe NRH	Obs ICU	Kampala	5	8
Mengo Hospital	GICU	Kampala	4	6
Norvik Hospital	GICU	Kampala	4	4

ICU - intensive care unit; NRH - national referral hospital; GICU - general intensive care unit; RRH - regional referral hospital; MICU - medical intensive care unit; MSWNH - Mulago Specialized Womens and Neonatal Hospital; CVICU - cardiovascular intensive care unit.

This is an open access article under the CC BY license (https://creativecommons.org/licenses/by/4.0/).

MOTIVATE-ICU eCRF ICU registry platform variables

Data points - MOTIVATE additions in blue; ICRU in black

Core variables

These variables provide essential information to enable reporting of case mix, acuity, and risk adjusted outcome. The patient identifiers are used to enable individual intensive care unit (ICU) stakeholders to track patients during the encounter. These are removed during data curation and an encounter ID is generated. Variables in bold are the parent variable, subsequent non bold variables provide greater granularity.

Table 2SA - Admission data

Variable name	Data entry format	Definitions for collection
Study ID*	Alphanumeric	Automatically generated based on study site
Medical record number ID*	Alphanumeric	Hospital number given to the patient for specific hospital encounters or repeated encounters
Age *P**	Years	Chronological age on the day of ICU admission
Sex *P**	Options: male, female, intersex	Sex at birth
Contact number *ID*†	Numerical	Phone number of patient or NOK that can be used to contact
Date of hospital admission *P**	yyyy/mm/dd	Date of arrival in the hospital
Time of hospital admission *P**	hh:mm:ss	Time of arrival in the hospital
Date of ICU admission *P**	yyyy/mm/dd	Date of arrival in the ICU
Time of ICU admission *P**	hh:mm:ss	Time of arrival in the ICU
ICU admission source *P**	Dropdown list	Patient's clinical location prior to ICU admission
Readmission	Options: yes or no	Patient discharged from ICU and readmitted during the same hospital admission
Date of previous discharge†	yyyy/mm/dd	If readmission is yes;<br>Previous ICU discharge date during same hospital admission
Type of admission *P**	Options: nonoperative, postoperative	Nonoperative - Admissions not arriving directly from OR or PACU including patients who may have undergone on operative procedure earlier in their hospital admission<br>Postoperative - Admission directly from the OR or PACU
Emergency surgery *P*†	Options: yes or no	If type of admission is postoperative;<br>Immediate surgery, where resuscitation (stabilization and physiological optimization) is simultaneous with surgical treatment and where surgery normally takes place within minutes of decision to operate or as deemed by surgical team
Reason for admission (operative) *P**	Type to search	If postoperative admission;<br>Search for the operative procedure from the SNOMED CT1 list which most accurately describes what procedure was undertaken. If there have been multiple operative procedures, use the additional fields to add additional procedures
Reason for admission (disorder) *P*†	Type to search	If postoperative admission;<br>In addition to the operative procedure, search and select any disorders from the SNOMED CT list which necessitated admission to the ICU.
Reason for admission (disorder) *P**	Type to search	If nonoperative;<br>The main disorder which has necessitated admission to the ICU. This should be based only on what is known or suspected to be primary disorder within the first hour of admission. Any additional disorders deemed to be significant to the patient's admission to the unit should be added using the additional reason for admission fields.
SARI diagnosis *S**	Options: confirmed. suspected, no	Indicate if at discharge the patient has a clinically suspected or laboratory confirmed severe acute respiratory infection as per WHO definition
If SARI confirmed or suspected - organism†	Dropdown list	If SARI is suspected or confirmed;<br>Specify the organism identified as precisely as possible.
Type of test†	Dropdown list	If SARI confirmed;<br>The type of test used to confirm the SARI diagnosis from a given list

Continue...

...continuation

Other type of test†	Text	If type of test is other;<br>Any other type of test used to confirm the SARI diagnosis
Comorbidities *P**	Dropdown list	Conditions currently present or diagnosed prior to this hospital admission (CCI)
Comorbidities (Other)†	Type to search	If comorbidities is other;<br>Search the SNOMED CT1 list and select any other conditions that are not available in the comorbidities dropdown list.
**Admission assessment**		
Screened for eligibility for MOTIVATE-ICU? *	Options: yes or no	Select whether the patient has been screened for inclusion in the study.
Age ≥ 15 years*	Options: yes or no	Record whether the patient is 15 years or older.
Transferred ≥ 24 hours after initiation of mechanical ventilation*	Options: yes or no	Record whether the patient was transferred in from non-study ICU after initiation of mechanical ventilation
Clinical frailty score (prior to critical illness)*	Dropdown list	*If enrolled in the MOTIVATE-ICU study*<br>Number 1 - 9 indicating physical functionality and level of independence prior to current hospital admission or current illness<br>1. Very fit (regularly exercises)<br>2. Fit (occasionally exercises)<br>3. Managing well (not active beyond routine walking)<br>4. Vulnerable (independent but limited outside activities)<br>5. Mildly frail (needs help with heavy housework)<br>6. Moderately frail (needs help with bathing)<br>7. Severely frail (completely dependent but not high risk of dying)<br>8. Very severely frail (completely dependent, at end of life)<br>9. Terminally ill (life-expectancy<6months, not otherwise evidently frail)
Immunosuppressive treatment*	Options: yes or no	*If enrolled in the MOTIVATE-ICU study*<br>Treatment known to cause immunosuppression; chemotherapy or steroids, AIDS
Height*	cm	*If enrolled in the MOTIVATE-ICU study*<br>Measured height or length in cm (may be estimated)
Ventilation*	Options: yes or no	Self-vent: No breaths are delivered by a mechanical device during the first hour of admission to ICU.<br>Mechanical vent: All or some of the breaths or a portion of the breaths (pressure support) are delivered by a mechanical device during the first hour of admission to ICU.
Route of ventilation*	Dropdown list	*If* mechanical vent*; options are:*<br>The type of mechanically assisted breathing during the first hour of admission. If multiple modes are used, please report the most invasive.<br>ETT<br>Tracheostomy<br>NIV mask
Route of ventilation*	Dropdown list	*If self-vent; options are:*<br>High flow oxygen (min flow 30/min)<br>Tracheostomy<br>Own airway
CPAP*	Options: yes or no	*If self-vent and enrolled in the MOTIVATE-ICU study*<br>Record whether the patient received CPAP.
Primary Indication of IMV* ‡	Dropdown list	*If enrolled in the MOTIVATE-ICU study*<br>Indications: upper airway protection, hypoxemic respiratory failure, hypercapnic respiratory failure, impending respiratory arrest, severe hemodynamic instability, cardiac arrest/post-cardiac arrest, post-anesthesia care, depressed level of consciousness
Mode of invasive ventilation* ‡	Dropdown list	PC-AC, VC-AC, PC-SIMV, VC-SIMV, PSV, PRVC, T-piece
Tidal volume* ‡	mL	*If enrolled in the MOTIVATE-ICU study*<br>Set tidal volume if volume mode<br>Measured expired tidal volume if pressure mode.<br>Enter the tidal volume recorded in the 1st hour of initiation of ventilation. If not available or applicable, then the value closest to 08:00hrs.

Continue...

...continuation

Peak inspiratory pressure/inspiratory pressure* ‡	cmH2O	*If enrolled in the MOTIVATE-ICU study*<br>Measured peak inspiratory pressure if volume mode<br>Set inspiratory pressure if pressure mode<br>Enter the pressure recorded in the 1st hour of initiation of ventilation. If not available or applicable, then the value closest to 08:00hrs.
FiO□ *P**	/ or %	The highest recorded inspired oxygen concentration during the first hour of admission to ICU. If not available in the first hour, then the highest value in the first 24 hours should be recorded.
SpO□ *P**	%	Percentage of oxygen-saturated hemoglobin during the first hour of admission to ICU. If not available in the first hour, the worst value in the first 24 hours should be recorded.
PaO□ *P*†	mmHg or KPa	The partial pressure of oxygen measured in the arterial blood. The lowest recorded during the first hour of admission. If not available in the first hour, the lowest value in the first 24 hours should be recorded.
Arterial pH *P*†	pH	The measurement of the pH of plasma of an arterial blood sample. The most deranged reading during the first hour of admission must be recorded. This is the reading furthest from the normal range of pH 7.35 to 7.45. If not available, the worst in the first 24 hours.
Respiratory rate *P**	b/min	Self-vent on admission: first recorded RR on admission to the ICU.<br>Mechanical vent on admission: last recorded RR prior to intubation.
Heart rate *P**	b/min	First recorded heart rate within the first hour of admission to the ICU
Systolic blood pressure *P**	mmHg	First recorded systolic BP within the first hour of admission to the ICU.
Diastolic blood pressure *P**	mmHg	First recorded diastolic BP within the first hour of admission to the ICU.
Temperature *P**	°F or °C	First recorded temperature within the first hour of admission to the ICU. If not available, the worst recorded value in the 1st 24 hours. Worst value being the farthest from the normal axillary range 36.0 - 37.5C or 96.8 - 99.5F
Sedated *P**	Options: yes or no	Use of sedative drugs for a minimum of one hour continuous infusion, or greater than one bolus.
Sedation medication*	Dropdown list	*If enrolled in the MOTIVATE-ICU study*<br>Enter the sedatives used at initiation of ventilation<br>Benzodiazepines<br>Ketamine<br>Propofol<br>Dexmedetomidine/Clonidine<br>Opioids
Sedation medication	Dropdown list	*If enrolled in the MOTIVATE-ICU study*<br>Enter the sedatives used at initiation of ventilation<br>Benzodiazepines<br>Ketamine<br>Propofol<br>Dexmedetomidine/Clonidine<br>Opioids
Sedation medication	Dropdown list	*If enrolled in the MOTIVATE-ICU study*<br>Enter the sedatives used at initiation of ventilation<br>Benzodiazepines<br>Ketamine<br>Propofol<br>Dexmedetomidine/Clonidine<br>Opioids
Glasgow coma scale (eye) *P**	Score 1 - 4	Self-vent on admission: the first recorded eye response for GCS score on admission to ICU.<br>Mechanical vent on admission: The last recorded eye response for GCS immediately prior to intubation.
Glasgow coma scale (verbal)*	Score 1 - 5	Self-vent on admission: the first recorded verbal response for GCS score on admission to ICU.<br>Mechanical vent on admission: the last recorded verbal response for GCS immediately prior to intubation.
Glasgow coma scale (motor)*	Score 1 - 6	Self-vent on admission: The first recorded motor response for GCS score on admission to ICU.<br>Mechanical vent on admission: The last recorded motor response for GCS immediately prior to intubation.
Jaundice*	Options: yes or no	*If enrolled in the MOTIVATE-ICU study*<br>Presence of conjunctival jaundice of any degree on admission or if not available, in the 1st 24 hours of admission.

Continue...

...continuation

**Antimicrobial use** *S**	Options: yes or no	Use of antimicrobial therapy. Even if only one dose has been administered this is considered as a 'yes'.
Antimicrobial type†	Dropdown list	If Antimicrobial use is yes;<br>The type of antimicrobial from a drug list.
Cardiovascular support *P**	Options: yes or no	Continuous intravenous;<br>inotropic or vasoactive medication within the first hour of ICU admission.
Vasoactive therapy†	Dropdown list	If Cardiovascular support is yes;<br>The type and dose of vasoactive drug used from the list of options.
**Renal replacement therapy** *P**	Options: yes or no	Use of renal replacement therapy. Any duration of RRT is considered as a 'yes'. Either in the 1st hour of admission or in the first 24 hours of admission.
Urine output*	Options:<br>$<$ 200mL/day<br>or 2, $<$ 500mL/day,<br>3 $\geq$ 500mL/day	*If enrolled in the MOTIVATE-ICU study*<br>Total urine output in the 1st 24 hours of ICU admission
Haemoglobin *P*	g/dL or g/L	Hb measured within the first hour of admission to ICU. If unavailable, provide the last reported Hb prior to admission (max 24 hours prior) or within first 24 hours of admission
Platelet *P*†	x10^9/L or K/μL<br>or 10 ^ 3/mm ^ 3<br>or x10 ^ 3/μL<br>or cells/μL<br>or lakhs/mm^3	Platelet count measured within the first hour of admission to ICU. If unavailable last reported platelet counts prior to admission (max 24 hours prior) or within first 24 hours of admission
Packed cell volume *P*†	% or /	Also called haematocrit. This is the volume percentage of red blood cells in blood measured within the first hour of admission to ICU. If unavailable, provide the last reported PCV prior to admission (max 24 hours prior) or within the first 24 hours of admission
Serum sodium *P*†	mEq/L or mmol/L	Blood sodium measured within the first hour of admission to ICU. If unavailable, provide the last reported blood sodium prior to admission (max 24 hours prior) or within the first 24 hours of admission
Serum potassium *P*†	mEq/L or mmol/L	Blood potassium measured within the first hour of admission to ICU. If unavailable, provide the last reported blood potassium prior to admission (max 24 hours prior) or within the first 24 hours of admission
Serum HCO3 *P*†	mEq/L or mmol/L	Blood bicarbonate measured within the first hour of admission to ICU. If unavailable, provide the last reported blood bicarbonate prior to admission (max 24 hours prior) or within the first 24 hours of admission
Serum creatinine *P*†	mg/dL or mmol/L or μmol/L or mg/L	Blood creatinine measured within the first hour of admission to ICU. If unavailable, provide the last reported blood creatinine prior to admission (max 24 hours prior) or within the first 24 hours of admission
Blood urea *P*†	mg/dL or mmol/L or g/L	Blood urea measured within the first hour of admission to ICU. If unavailable last reported blood urea prior to admission (max 24 hours prior) or within the first 24 hours of admission
White Blood cell count†	10 ^ 9/L or K/μL<br>or cells/mm^3<br>or 10 ^ 3/mm^3<br>or other	WBC measured within the first hour of admission to ICU. If unavailable, last reported measurement prior to admission (max 24 hours prior) or within the first 24 hours of admission.
Total Bilirubin	umol/L or mg/dL	Total Serum bilirubin measured within the first hour of admission to ICU. If unavailable, last reported measurement prior to admission (max 24 hours prior) or within the first 24 hours of admission.
Chest X-ray/CT*	Options: yes or no	*If enrolled in the MOTIVATE-ICU study*<br>Including chest CT-scan.<br>Done during 1st 24 hours of ICU admission
Chest X-ray/CT findings*	Dropdown list	*If enrolled in the MOTIVATE-ICU study*<br>*If 'Chest X-ray' or chest CT-Scan is 'yes':*<br>*Normal, New Infiltrates, Persistent infiltrates, Consolidation, Cavitation, Pneumothorax, Pulmonary edema, Atelectasis, Pleural effusion, Pulmonary embolism*
**Discharge**		
Date of ICU discharge *P**	yyyy/mm/dd	The date on which the patient is discharged from or dies in the ICU.
Time of ICU discharge *P*†	hh:mm:ss	The time on which the patient is discharged from or dies in the ICU.

Continue...

...continuation

ICU discharge status *P**	Dropdown list	Whether the patient is alive at the point of ICU discharge.
Enrolled in MOTIVATE-ICU study?*	Options: yes or no	Record whether the patient has been enrolled in the MOTIVATE-ICU study
Family/ lay care provider in ICU	Options: yes or no	*If enrolled in the MOTIVATE-ICU study and consent was withdrawn*<br>Record whether the patient had a family or family appointed lay care provider whilst in the ICU, who assisted with daily care activities
Consent withdrawn for MOTIVATE-ICU??*	Options: yes or no	*If enrolled in the MOTIVATE-ICU study and consent was withdrawn*<br>Record whether the patient has been withdrawn from the MOTIVATE-ICU study.
Date withdrawn*	DateCor	*If enrolled in the MOTIVATE-ICU study and consent was withdrawn*<br>Record the date the patient/legal representative withdrew consent. Consent must be withdrawn within 72 hours of ICU admission.
Time withdrawn*	Time	*If enrolled in the MOTIVATE-ICU study and consent was withdrawn*<br>Record the time the patient/legal representative withdrew consent. Consent must be withdrawn within 72 hours of ICU admission.
Primary Indication for tracheostomy*	Dropdown list	*If enrolled in the MOTIVATE-ICU study and consent was not withdrawn*<br>Weaning failure<br>Anticipated prolonged MV<br>Prolonged MV<br>Upper airway obstruction<br>Airway protection<br>Not known.<br>No tracheostomy performed
Tracheostomy type*	Dropdown list	*If enrolled in the MOTIVATE-ICU study and consent was not withdrawn and 'primary indication for tracheostomy' is not 'no tracheostomy performed'*<br>surgical trach<br>Percutaneous trach
Tracheostomy complications*	Dropdown list	*If enrolled in the MOTIVATE-ICU study and consent was not withdrawn*<br>SSI<br>Major bleeding<br>Dislodgement<br>Malfunction<br>None
Decannulation date	yyyy/mm/dd	*If enrolled in the MOTIVATE-ICU study and consent was not withdrawn*<br>Date the tracheostomy was decannulated.
End-of-life care and/or ICU palliative support*	Options: yes or no	*If enrolled in the MOTIVATE-ICU study and consent was not withdrawn*<br>Record whether the patient was admitted for end-of-life care and/or ICU palliative support or determined to be for end-of-life care
Date care became palliative*	Date	*If 'end-of-life care and/or ICU palliative support' is 'yes'*<br>Record the date the patient was determined to be for end-of-life care and/or ICU palliative support.

* failure to save alert; † pre saving alert; ‡ If on invasive mechanical ventilation.

Admission
P = used to calculate standardized mortality ratio. Captured close to time admission, calculated using E TropICS or APACHE II.
ID = used to identify encounters during the inpatient episode. Replaced with a unique platform encounter ID once encounter completes. S = used for infection surveillance.

References:
1. Donnelly K. SNOMED-CT: The advanced terminology and coding system for eHealth. Stud Health Technol Inform. 2006;121:279-90.
2. ICNARC Case Mix Programme Data Collection Manual v3.1.
3. Ho KM, Dobb GJ, Knuiman M, Finn J, Lee KY, Webb S. A comparison of admission and worst 24-hour Acute Physiology and Chronic Health Evaluation II scores in predicting hospital mortality: a retrospective cohort study. Crit Care. 2006;10(1):R4.

Table 2SB - Daily variables

**Daily Q Assessment<br>Completed every day for the inpatient ICU stay up to 28 days. Data reflects events for the previous 24 hours: 00:00 - 24:00hrs.**		
**Care of the ventilated patients**		
**Variable name**	**Data entry format**	**Definition for collection**
Enrolled in MOTIVATE-ICU study?*	Options: yes or no	Record whether the patient has been enrolled in the MOTIVATE-ICU study.<br>If the patient was not eligible, or the patient/representative withdrew consent within 72 hours of admission, please select 'no'.
Ventilation*	Options: yes or no	Self-vent: no breathing is delivered by a mechanical device for a period of one hour during the day.<br>Mechanical vent: all or some of the breaths or a portion of the breaths (pressure support) are delivered by a mechanical device for one hour during the day. Please record the highest ventilation support in the last 24 hours
Route of ventilation*	Dropdown list	*If mechanical vent; options are:*<br>The type of mechanically assisted breathing during the first hour of admission. If multiple modes are used, please report the most invasive.<br>ETT<br>Tracheostomy<br>NIV mask
Route of ventilation*	Dropdown list	*If* self-vent; *options are:*<br>High flow oxygen (min flow 30/min)<br>Tracheostomy<br>Own airway
CPAP*	Options: yes or no	*If self-vent and enrolled in the MOTIVATE-ICU study*<br>Record whether the patient received CPAP.
Primary Indication of IMV* ‡	Dropdown list	*If enrolled in the MOTIVATE-ICU study*<br>Indications: upper airway protection, hypoxemic respiratory failure, hypercapnic respiratory failure, impending respiratory arrest, severe hemodynamic instability, cardiac arrest/post-cardiac arrest, post-anesthesia care, depressed level of consciousness
Mode of invasive ventilation* ‡	Dropdown list	PC-AC, VC-AC, PC-SIMV, VC-SIMV, PSV, PRVC, T-piece
Tidal volume* ‡	ml	*If enrolled in the MOTIVATE-ICU study*<br>Set tidal volume if volume mode<br>Measured expired tidal volume if pressure mode.<br>The tidal volume closest to 10am should be recorded.
Peak inspiratory pressure/ inspiratory pressure* ‡	cmH2O	*If enrolled in the MOTIVATE-ICU study*<br>Measured peak inspiratory pressure if volume mode<br>Set inspiratory pressure if pressure mode<br>The peak inspiratory pressure/inspiratory pressure closest to 10am should be recorded.
Tracheal secretions*	Dropdown list	*If enrolled in the MOTIVATE-ICU study*<br>*If 'mechanical vent' is yes;*<br>*Appearance of ETT/TT secretions*<br>*— Purulent*<br>*— Non-purulent*<br>*— Minimal/none*
Spontaneous breathing trial (SBT)	Options: yes or no	*If 'mechanical vent' is yes: and*<br>*'Route of ventilation' is 'tracheostomy': or 'ETT':*<br>A trial of spontaneous breathing, defined as one of the following four;<br>— Trial of 'T' piece<br>— Pressure support with low support (PS of 5 - 8)<br>— CPAP (i.e. only PEEP between 0 - 5)<br>— Automatic tube compensation with CPAP<br>— Not eligible for SBT
Lowest FiO_2	% or ratio ≤ 1.0	*If 'mechanical vent' is yes:*<br>Lowest inspired oxygen concentration delivered for a minimum of one hour for the last 24 hours
Lowest PEEP	mmH_2O	*If 'mechanical vent' is yes:*<br>Lowest positive end expiratory pressure for a minimum of one hour for the last 24 hours
SpO_2	%	The lowest peripheral oxygen saturation measured by pulse oximetry in the last 24 hours.

Continue...

...continuation

Respiratory rate highest	b/min	The highest measured respiratory rate recorded in the last 24 hours. Either set RR if no spontaneous breathing or total respiratory rate (set RR + spontaneous RR)
VTE prophylaxis	Dropdown list	*If 'mechanical vent' is yes:*<br>Use of mechanical or pharmacological prophylaxis in the prevention of venous thromboembolism.
Contraindication	Dropdown list	*If 'VTE prophylaxis' is 'none':*<br>The contraindication preventing the use of mechanical and pharmacological prophylaxis.
Stress ulcer prophylaxis	Options: yes or no	*If 'ventilation' is 'mechanical vent':*<br>A drug is prescribed for the purpose of stress ulcer prophylaxis
Drug	Dropdown list	*If 'stress ulcer prophylaxis' is 'yes':*<br>The named drug from the provided list used for stress ulcer prophylaxis.
*Inadvertent/Accidental extubation**	*Options: yes or no*	*If 'mechanical vent' is yes:*<br>*The unexpected or unplanned extubation of a patient requiring reintubation*.
Suspected or confirmed vent tube blockage*	Options:<br>1. yes, and tube replaced<br>2. yes, and patient extubated/ decannulated,<br>3. no	*If 'mechanical vent' is yes:*
Head of bed >30° or patient sat up (if no degree measure)*	Options: yes or no	*If 'mechanical vent' is yes:*<br>*Position of the head of bed*<br>Please record head of bed position daily based on observed elevation at time of review.<br>If no degree measures, patients have hip flexion with chest and head elevated using pillows or have total bed tilt (Trendelenburg).
Chest X-ray*	Options: yes or no	Chest X-ray done in the last 24 hours
Chest CT*	Options: yes or no	*If enrolled in the MOTIVATE-ICU study*<br>Chest CT done in the last 24 hours
Chest X-ray/CT findings*	Dropdown list	*If enrolled in the MOTIVATE-ICU study*<br>*If 'chest X-ray' or chest CT-scan is 'yes':*<br>*Normal, new infiltrates, persistent infiltrates, consolidation, cavitation, pneumothorax, pulmonary edema, atelectasis, pleural effusion, pulmonary embolism*
Pulmonary complications*	Dropdown list	*None, new infiltrates, persistent infiltrates, pneumothorax, pleural effusion, atelectasis , development of ARDS, cardiogenic pulmonary edema, pulmonary infection*
**CVS care**		
Highest temperature	°F or °C	Highest recorded temperature
Cardiovascular support	Options: yes or no	Use of continuous intravenous inotropic and/or vasoactive medication for a minimum period of one hour.
Vasoactive drugs increased	Options: yes or no	*If 'cardiovascular support' is 'yes':*<br>An increase in the use of vasoactive medication (either number of agents or dosage of an agent) for a minimum period of one hour.
Indwelling devices	Options: new, *insitu* or no	The presence of an indwelling catheter<br>Catheter types<br>– Central venous catheter<br>– Hemodialysis catheter<br>– Arterial catheter<br>– Peripheral cannula<br>– Urinary catheter<br>– Chest drain<br>– Abdominal (or other drain).
Site	Dropdown list	*If 'central venous catheter', 'arterial catheter' or 'peripheral cannula' or 'urinary catheter' is 'new' or 'in situ':*<br>Select the location of the new or existing central venous catheter, arterial catheter or peripheral cannula.
Daily cumulative fluid balance*	Numerical (-10000 to +10000mL)	*If enrolled in the MOTIVATE-ICU study*<br>Recorded from the patient fluid monitoring chart record from 1st 24 hours
**Renal care**		
**Renal replacement therapy**	Options: yes or no	Use of renal replacement therapy. Any duration of renal replacement therapy is considered as 'yes'.

Continue...

...continuation

Type of the therapy	Dropdown list	*If 'renal replacement therapy' is 'yes':*<br>The type of renal replacement therapy used.
**Neuro care**		
**Sedated**	Options: yes or no	Use of sedative drugs for a minimum of one hour. If bolus sedation, then 2 or more bolus.
Sedation medication*	Dropdown list	*If enrolled in the MOTIVATE-ICU study & yes to sedated*<br>Enter the sedatives used at initiation of ventilation<br>Benzodiazepines<br>Ketamine<br>Propofol<br>Dexmedetomidine/Clonidine<br>Opioids
Sedation medication	Dropdown list	*If enrolled in the MOTIVATE-ICU study & yes to sedated*<br>Enter the sedatives used at initiation of ventilation<br>Benzodiazepines<br>Ketamine<br>Propofol<br>Dexmedetomidine/clonidine<br>Opioids
Sedation medication	Dropdown list	*If enrolled in the MOTIVATE-ICU study & yes to sedated*<br>Enter the sedatives used at initiation of ventilation<br>Benzodiazepines<br>Ketamine<br>Propofol<br>Dexmedetomidine/Clonidine<br>Opioids
Sedation medication	Dropdown list	*If enrolled in the MOTIVATE-ICU study & yes to sedated*<br>Enter the sedatives used at initiation of ventilation<br>Benzodiazepines<br>Ketamine<br>Propofol<br>Dexmedetomidine/Clonidine<br>Opioids
Spontaneous awakening trial or (sedation hold).	Options: yes or no	*If 'sedated' is 'yes':*<br>Spontaneous awakening trial (SAT or "sedation hold") a period during which sedating medications that are being used to treat the patient were held in order to determine whether the patient requires ongoing sedation or can be managed without sedatives.
**Target RASS**	Dropdown list	*If 'mechanical vent' is yes and 'sedated' = yes*<br>The target RASS is a measure of sedation, awakeness and agitation. The target RASS is the target set each day on the ward round.<br>The options are available as a drop down.<br>*+4 to -5 and 'no target set'*
**Actual RASS score**	Dropdown list	*If 'ventilation' is 'mechanical vent' and 'sedated' = yes*<br>RASS is a measure of sedation, awakeness and agitation.<br>The actual RASS is the measured (observed) `RASS either immediately prior to a SAT or between 0800 -1200 hours.<br>The options are available as a drop down.<br>*+4 to -5 and 'not recorded'*
**Skin care**		
**Pressure injury**	Options: yes or no	Pressure/ compression related injury to skin
Site of pressure injury	Dropdown list	*If 'pressure injury' is 'yes':*<br>– Back of the head<br>– Ears, face, mouth<br>– Shoulders<br>– Elbows<br>– Lower back<br>– Buttocks/ sacrum<br>– Hips<br>– Inner knees<br>– Heels<br>– Other

Continue...

...continuation

Grade	Dropdown list	*If 'pressure injury' is 'yes':* 1, 2, 3, 4, 5
**Infection management**		
**Highest white cell count**	$x10^9$/L or $x10^3$/mm^3 or $x10^3$/μL	The highest recorded serum white blood cell count.
**Lowest white cell count**	$x10^9$/L or $x10^3$/mm^3 or $x10^3$/μL	The lowest recorded serum white blood cell count.
**Infection**	Dropdown list	Presence of a confirmed or suspected infection.
Source	Dropdown list	*If 'infection' is 'suspected' or 'confirmed':*<br>The source of infection from a list of body systems.
Clinician treating as healthcare associated infection	Options: yes or no	Clinicians may be treating patients for healthcare associated infection with or without microbiological confirmation. Information used to help interpret abx choice and perceived incidence of HCAI.
**Antimicrobial use daily**	Options: yes or no	Use of antimicrobial therapy. Even if only one dose has been administered, this is 'yes'<br>Denominator- all ICU patients<br>Eligibility = all.
Antimicrobial type	Dropdown list	*If 'Antimicrobial use daily' is 'yes':*<br>The type of antimicrobial from a drug list.
**Microbiology**		
**Culture obtained today**	Options: yes or no	Was a culture sample taken today.
Type of culture	Dropdown list	Type of culture.<br>*1, Blood | 2, Urine | 3, CSF | 4, Stool | 5, Sputum | 6, BAL | 7, Wound*
**Culture report**	Dropdown list	*1, Growth | 2, No growth | 3, Pending | 4, None Obtained | 5, None Pending*<br>Record whether a new culture report was available today.<br>Growth: if a new culture report was available today and growth was identified.<br>No growth: if a new culture report was available today and no growth was identified.<br>Pending: if a culture report was obtained and results are pending<br>None obtained: if no cultures have been obtained during the patient's say<br>None pending: if results of all cultures taken are available and have been previously entered on the patient's record.
Date culture taken	yyyy/mm/dd	*If 'culture report' is 'growth' or 'no growth':*<br>Record the date the specimen was taken.
Organism	Dropdown list	*If 'culture report' is 'growth':*<br>Organism list referred by HELICS (Note: refer Organism list sheet)
Resistance name	Dropdown list	*If 'Organism' is recorded:*<br>From the dropdown list, record the antimicrobial resistance for the organism
Sensitivity name	Dropdown list	*If 'Organism' is recorded:*<br>From the dropdown list, record the antimicrobial sensitivity for the organism
**Mobilization and rehabilitation**		
Physiotherapy/Mobilisation	Dropdown list	*If 'mechanical vent' is yes*':<br>Patients ventilated may receive passive or active physiotherapy in the ICU. The type of physiotherapy provided should be recorded daily. The event may occur at any time in the previous 24 hours. Some patients may not be eligible for daily physio (for example due to cardiovascular instability, or because they are at the end of life or because they are receiving a procedure).<br>*1. On bed physiotherapy*<br>*2. Out of bed mobilisation*<br>*3. No physiotherapy delivered*<br>*4. No physiotherapy delivered due to patient not eligible*

* failure to save alert; ‡ If on invasive MV.

References:
1. Donnelly K. SNOMED-CT: The advanced terminology and coding system for eHealth. Stud Health Technol Inform. 2006;121:279-90.
2. ICNARC Case Mix Programme Data Collection Manual v3.1.
3. Ho KM, Dobb GJ, Knuiman M, Finn J, Lee KY, Webb S. A comparison of admission and worst 24-hour Acute Physiology and Chronic Health Evaluation II scores in predicting hospital mortality: a retrospective cohort study. Crit Care. 2006;10(1):R4.

Table 3S - Modified SOFA score

SOFA score	0	1	2	3	4
Respiratory*					
PaO_2/FiO_2	> 400	300 - 400	200 - 299	100 - 199	≤ 100
SpO_2/FiO_2		316 - 400	236 - 315	151 - 235	≤ 150
Coagulation (platelets $10^3/mm^3$)	> 150	< 150	< 100	< 50	< 20
Liver					
Bilirubin (mg/dL)	< 1.2	1.2 - 1.9	2.0 - 5.9	6.0 - 11.9	> 12.0
Scleral icterus/Jaundice*	No			Yes	
Cardiovascular					
Hypotension (mcg/kg/min)	No hypotension	MAP < 70	Dobutamine (any dose)	Norepinephrine/ epinephrine ≤ 0.1	Norepinephrine/ epinephrine > 0.1
CNS					
Glasgow coma score	15	13 -14	10 - 12	6 - 9	< 6
Renal					
Creatinine (mg/dL)	< 1.2	1.2 - 1.9	2.0 - 3.4	3.5 - 4.9	> 5.0
Urine output (mL/d)				< 500	< 200

SOFA - Sequential Organ Function Assessment score; PaO_2 - partial pressures of oxygen in plasma; SpO_2 - peripheral saturation of oxygen; FiO_2 - fraction of inspired oxygen; CNS - central nervous system. * Modified to include SpO_2/FiO_2 ratio, clinical jaundice for the liver dysfunction if no blood gas analysis or Liver function tests.
Reference: Rahmatinejad Z, Reihani H, Tohidinezhad F, Rahmatinejad F, Peyravi S, Pourmand A, et al. Predictive performance of the SOFA and mSOFA scoring systems for predicting in-hospital mortality in the emergency department. Am J Emerg Med. 2019;37(7):1237-41.

Table 4S - Electronic Case Report Form for intensive care unit organizational characteristics for Redcap

**Baseline information**	
1. Hospital name	Open text
2. Location (dropdown)	a. Kampala<br>b. Gulu<br>c. Mbarara<br>d. Mbale<br>e. Jinja
3. Designation of the person providing the information (dropdown menu)	a. Consultant ICU<br>b. Fellow ICU<br>c. Resident ICU<br>d. Medical officer<br>e. Nursing in-charge<br>f. Nurse, Technician
4. Contact e-mail	Open text

**Hospital characteristics**<br>Please, provide the following information that describes your hospital MOST appropriately:	
**Variable**	**Data to be collected**
1. Type of hospital (dropdown menu)	Public<br>PNFP<br>Private
1.1 University-affiliated?	Yes/No
2. Number of hospital beds (i.e., active hospital beds) (estimate if not certain)	Open text
3. Total number of intensive care units in the hospital (including cardiac and coronary care, but excluding intermediate, and step-down units)<br><br>Intensive care unit definition: intensive care unit as a designated unit within a hospital that routinely provides invasive mechanical ventilation therapy with continuous vital sign monitoring (electrocardiographic monitoring, heart/pulse rate, noninvasive blood pressure, peripheral oxygen saturation) and designated nursing care for each bed in the unit i.e, at least three patients per week for at least 24 hours.	Open text
4. Is (are) there intermediate or step-down unit(s)?	Yes/No
5. Is there an Emergency Room or Department? (dropdown)	No<br>Yes, open<br>Yes, referenced
6. Is the hospital certified by an Accreditation Organization? (dropdown)	No<br>Yes, national<br>Yes, international
7. Is there a hospital wide rapid response team formally implemented for $\geq$ 6 months? (dropdown)	Yes/No
8. Are there training programs or medical residence at your institution? (dropdown)	No<br>Yes, but not in critical care<br>Yes, including critical care
9. Are there training programs for graduate nurses at your institution? (dropdown)	No<br>Yes, but not specific to critical care<br>Yes, including specific training in critical care
10. Are there training programs for other care providers (physiotherapists, psychologists, clinical pharmacists or nutritionists) at your institution?	No<br>Yes, but not specific to critical care<br>Yes, including specific training in critical care

**Intensive care unit characterization form**<br>Please, provide the following information that describes your intensive care unit MOST appropriately. If there is more than one intensive care unit at your hospital, please, fill an "intensive care unit form" for each of them	
Intensive care unit name:	Open text

I - ICU characterization	
1. ICU type (dropdown)	General or mixed medico-surgical; surgical; medical; neurological; coronary or cardiac; other
2. How long has your ICU been functional?	Open text
3. Number of active ICU beds<br>*(Beds used for continuous monitoring, mechanical ventilation and other organ support services)*	Open text
4. Estimated number of ICU admissions in past one year	Open text
5. Organ support capacity in addition to invasive mechanical ventilation within the ICU (checklist)	— Pharmacological cardiovascular support (inotropes and vasopressors)<br>— IABP<br>— ECMO<br>— Blood gas analysis<br>— Intermittent renal replacement therapy<br>— Continuous renal replacement therapy<br>— Parenteral nutrition
6. Organ monitoring capacity in addition to noninvasive monitoring within the ICU (checklist)	— CVP<br>— ABP<br>— Invasive cardiac output monitoring<br>— Pulmonary artery catheterization<br>— Invasive and/or noninvasive cerebral monitoring (NIRS, ICP monitoring, EEG)<br>— In-house ultrasonography monitoring

II - Staffing patterns: clinicians	
7. ICU model type<br>*Open unit: primary doctors make all care-related decisions in the ICU with the ICU doctor on consult as needed.*<br>*Closed unit: ICU doctor makes all care-related decisions.*<br>*Semi-closed: shared decision making between ICU doctor and primary doctor*	— Open unit<br>— Closed unit<br>— Semi-closed
8. Primary speciality of the consultant in charge of the ICU? (dropdown)	— Anesthesiology, Internal Medicine, Emergency medicine, Surgery, Pulmonology, Cardiology, Nephrology, Non-specialized
9. Is the Consultant in charge of ICU an intensivist (trained & certified in ICM for at least one year)?	— Yes/No
9.1 If no, is the consultant in charge of ICU a non-intensivist physician but with $\geq$ 2 years of ICU clinical experience?	— Yes/No
10. Which of the following specialists are available onsite in the hospital for ICU consultation? (checklist)	— Anesthesiologist, Cardiologist, Nephrologist, General surgeon, General physician, Pulmonologist, Infectious disease, Microbiologist, Obstetrician-gynecologist, Gastroenterologist, Neurologist, Hematologist, Pediatrician
11. Total number of ICU doctors in your ICU	— Open text
12. Qualification of ICU doctors employed in the ICU? (yick all that apply)	— Anesthesiology, Internal Medicine, Emergency medicine, Surgery, Pulmonology, Cardiology, Nephrology, Non-specialized, Specialist trainees (Specify - Anesthesia, EM, IM, Surgery), Medical officers
13. Do you have staff Intensivists in your ICU?<br>(Intensivist = Base specialty in IM, Anesthesia, EM, Surgery with $\geq$1-year certified training in Intensive care medicine)	— No/Yes

13.1 If yes, what is the most frequent coverage pattern on weekdays?	1. 24/7 in-house<br>2. Daytime with nighttime on-call<br>3. Daytime with nighttime anesthesiologist<br>4. Daytime with nighttime non-anesthesia specialist<br>5. Daytime with nighttime trainee<br>6. Daytime with nighttime medical officer<br>7. Daytime with nighttime AHP<br>8. Tele-ICU
13.2 If yes, what is the most frequent coverage pattern on weekends?	1. 24/7 in-house<br>2. Daytime with nighttime on-call<br>3. Daytime with nighttime anesthesiologist<br>4. Daytime with nighttime non-anesthesia specialist<br>5. Daytime with nighttime trainee<br>6. Daytime with nighttime medical officer<br>7. Daytime with nighttime AHP<br>8. Tele-ICU<br>9. None
14. Do you have staff non-intensivist specialty physicians in your ICU with at least 2 years' ICU clinical experience?	9. No/Yes
14.1 If yes, what is the most frequent coverage pattern on weekdays?	1. 24/7 in-house<br>2. Daytime with nighttime anesthesiologist<br>3. Daytime with nighttime non-anesthesia specialist<br>4. Daytime with nighttime trainee<br>5. Daytime with nighttime medical officer<br>6. Daytime with nighttime AHP<br>7. Tele-ICU
14.2 If yes, what is the most frequent coverage pattern on weekends?	1. 24/7 in-house<br>2. Daytime with nighttime anesthesiologist<br>3. Daytime with nighttime non-anesthesia specialist<br>4. Daytime with nighttime trainee<br>5. Daytime with nighttime medical officer<br>6. Daytime with nighttime AHP<br>7. Tele-ICU<br>8. None

Staffing patterns: nurses	
15. Is the ICU In-charge nurse certified in Intensive Care nursing from an accredited program?	Yes/No
15.1. If the incharge nurse isn't certified in Intensive care nursing; does he/she have ≥ 2 years of ICU clinical experience?	Yes/No
16. Total Number of nurses working in the ICU	Open text
17. Number of nurses certified in Intensive care nursing	Open text
18. Number of nurses (not certified) with ≥ 2 years of ICU clinical experience	Open text
19. Maximum number of self-ventilated patients 1 nurse looks after during the day (n)	Open text
20. Maximum number of ventilated patients 1 nurse looks after during the day (n)	Open text
21. Maximum number of self-ventilated patients 1 nurse looks after during the night (n)	Open text
22. Maximum number of ventilated patients 1 nurse looks after during the night (n)	Open text
23. Maximum number of self-ventilated patients 1 nurse looks after during the weekend (n)	Open text
24. Maximum number of ventilated patients 1 nurse looks after during the weekend (n)	Open text
25. Are there critical care trained nurses present in the ICU during night shift?	Yes/No
26. Are there critical care trained nurses present in the ICU during weekends?	Yes/No

Staffing patterns: physiotherapists and respiratory therapists (* excluding trainees and residents)	
27. Does the ICU team have access to physiotherapists?	No; yes, but not dedicated to the ICU; yes, dedicated to the ICU
27.1 In case of dedicated physiotherapists, they are present in the ICU	Only during day shifts; during day and night shifts

Staffing patterns: other care providers (* excluding trainees and residents)	
28. Nutritionist	No; yes, but not dedicated to the ICU; yes, dedicated to the ICU
29. Clinical pharmacist	No; yes, but not dedicated to the ICU; yes, dedicated to the ICU

III - Empowerment assessment of the multidisciplinary team

Are your ICU healthcare providers allowed to proceed with the following activities WITHOUT THE NEED of direct discussion with the intensive care physician, except in exceptional cases:

Check:

No: the healthcare provider CANNOT proceed with the activity without explicit communication to the ICU physician.

Sometimes: the healthcare provider CAN SOMETIMES proceed with the activity without explicit communication to the ICU physician in certain occasions or patients.

Yes: the healthcare provider CAN ALWAYS proceed with the activity without explicit communication to the ICU physician and is at his/her discretion to judge if the intensivist must be communicated in advance.

Nurses (*except residents and postgraduate students)	
30. Pause or titrate sedation, according to routine or protocol<br>31. Increase diet infusion, according to routine or protocol<br>32. Titrate vasopressors, directed to or based on goals<br>33. Start ventilatory weaning, according to routine/protocol<br>34. Increase FiO_2 (by either invasive or noninvasive device)<br>35. Start out of be active mobilization, according to routine/ protocol<br>36. Offer symptomatic prescribed as "if necessary" (SOS)<br>37. Provide patient information to the family/next of kin	No; sometimes; yes

Physiotherapists (*except residents and postgraduate students)	
38. Start ventilatory weaning, according to routine/protocol<br>39. Modify ventilator parameters and FiO_2, including PEEP<br>40. Increase or decrease oxygen support, including changing noninvasive devices (e.g. changing nasal catheter to Venturi mask)<br>41. Start noninvasive ventilation or high-flow oxygen nasal cannula<br>42. Perform alveolar recruitment maneuver<br>43. Start routine passive mobilization<br>44. Start active mobilization (including out of bed), according to routine/ protocol, including in ventilated patients	No; sometimes; yes

IV - Organizational aspects and patient care processes

Multidisciplinary rounds	
45. Do the rounds occur at the bedside?	No; sometimes; yes, always
46. Are multidisciplinary clinical rounds performed?	No<br>Yes, on some days of the week;<br>Yes, every day except weekends;<br>Yes, every day, including weekends
46.1 If yes, which professionals participate in multidisciplinary rounds? (check all that apply)	Physicians, nurses, physiotherapists, Psychologists, clinical pharmacist, nutritionists

Checklists	
47. Are checklists used during clinical rounds to assist with patient care and management?	No<br>Yes, on some days of the week;<br>Yes, daily, except weekends;<br>Yes, daily, including weekends
47.1. If yes, what are the functions of checklists in clinical rounds?	Only monitor adherence to best practices;<br>Monitor and guide the implementation of adherence to best practices

Continuity assurance procedures of patient care information during handover	
48. What is the handover model in your ICU?	Individual, each team of health professionals do the handover separately;<br>Multidisciplinary, all health professionals in the ICU do the handover together
49. Is there STRUCTURED formal documentation of the handover in written record?	Yes/No
49.1 If yes, what avenue is used? (check all that apply)	Form or template on paper<br>Electronic media
49.1.1 If yes, specify	Open text

V - Organizational aspects and patient care processes

Clinical protocols	
In the last 6 months, clinical care protocols are being implemented in your ICU?<br>**Protocol** is defined as an explicit, standardized and detailed written plan or clinical pathway that provides a set of guiding rules for caring for patients with a given condition.<br>**Check:**<br>No: no protocol OR protocol not implemented<br>Partially implemented: the protocol was elaborated, but the teams have not been adequately trained and there is no regular monitoring of indicators.<br>Fully implemented: the protocol has been elaborated and is fully implemented, with adequate structure, processes, actions, strategies, team training, as well as the regular monitoring and reporting of the indicators.	
50. Sedation (i.e., daily interruption of sedation or protocol- directed sedation in ventilated patients)	No; partially; fully
51. Liberation from mechanical ventilation (i.e., health care-driven spontaneous breathing trials)	No; partially; fully
52. Protective ventilation (i.e., ventilation with low tidal volumes in patients with acute lung injury or ARDS)	No; partially; fully
53. Fluid management protocols (i.e., implementation of conservative fluid management protocols including diuresis)	No; partially; fully
54. Prevention of catheter-related bloodstream infection (i.e., implementation of checklists during insertion and maintenance of intravascular catheters)	No; partially; fully
55. Prevention of ventilator-associated pneumonia VAP (i.e., implementation of best practices for the prevention of VAP in ventilated patients)	No; partially; fully
56. Early mobilization of ventilated patients (i.e., protocoled early mobilization and exercise (including out-of- bed exercises), including physical and occupational therapy during periods of sedation interruption in ventilated patients)	No; partially; fully
57. Hand-washing protocol (i.e,, soap and water as well as alcohol-hand rub during patient care)	No; partially; fully

Table 5SA - Intensive care unit classification scoring matrix

	Level 1 score	Level 2 score	Level 3 score	Weight
**Criteria**				
ICU model-type	Open	Semi-closed	Closed	2
ICU director	0 (specialist[a] < 2 years OR non-specialist)	1 (specialist ≥ 2 years ICU clinical experience)	2 (certified intensivist)	3
Staffing: physician coverage				
24/7	0 (specialist < 2 years OR non-specialist)	1 (specialist ≥ 2 years ICU clinical experience)	2 (certified intensivist)	3
Day time	0 (specialist < 2 years OR non-specialist)	1 (specialist ≥ 2 years ICU clinical experience)	2 (certified intensivist)	3
Night time	0 (MO, AHP, on-call)	1 (non-anesthesia specialist, specialist trainee)	2 (anesthesiologist)	2
Staffing: medical specialists	0 (< 25% on-site)	1 (25 - 50% on-site	2 (> 50% on-site)	2
Staffing: nursing coverage				
ICU in-charge nurse	0 (< 2 years ICU clinical experience)	1 (≥ 2 years ICU clinical experience)	2 (certified ICU training)	3
Staffing: nursing experience	0 (< 50% nurses with ≥ 2 years ICU clinical experience)	1 (≥ 50% nurses with ≥ 2 years ICU experience)	2 (≥ 50% nurses with certified ICU training)	3
Staffing: patient-to-nurse ratio	0 (≥ 3:1)	1 (≤ 2:1)	2 (1:1)	3
Allied health personnel				
Physiotherapist	0 (not available)	1 (available, not dedicated)	2 (dedicated to ICU)	2
Nutritionist	0 (not available)	1 (available, not dedicated)	2 (dedicated to ICU)	2
Clinical pharmacist	0 (not available)	1 (available, not dedicated)	2 (dedicated to ICU)	2
Monitoring				
Basic	0 (noninvasive)	1 (level 1 + invasive BP, CVP + blood gas analysis)	2 (level 2 + cardiac output, cerebral monitoring, in-house ultrasound)	3
Organ support: ventilation	0 (no IMV support, only non-invasive O_2 therapy)	1 (level 1 + intermittent RRT OR vasoactive support OR TPN)	2 (level 2 + ECMO OR CRRT OR IABP)	3
Rounds				
MDT	No MDT round	Only physicians + nurses < 5 days/week	Physician & nurses + physiotherapists OR nutritionist OR clinical pharmacist ≥ 5 days/week	3
Integration with ICU outreach				
Rapid Response Team	0 (none)		Formal RRT present	2
HDU/stepdown unit	0 (none)		Present	2
Quality improvement				
Clinical protocol implementation	0 (*ad hoc* OR no protocols OR no implemented protocols OR < 50% partial implementation OR < 25% full implementation)	1 (50 - 75% partial implementation OR 25 - 50% full implementation)	2 (full implementation of > 50% OR > 75% partial implementation)	3

ICU - intensive care unit; MO - medical officer; AHP - allied health personnel; BP - blood pressure; CVP - central venous pressure; IMV - invasive mechanical ventilation; RRT - renal replacement therapy; TPN - total parenteral nutrition; ECMO - extracorporeal membrane oxygenation; CRRT - continuous renal replacement therapy; IABP - intraaortic balloon pump; RRT - Rapid Response Team; HDU - high dependency unit. [a] Specialist - graduate from a formal specialist training training program after first medical degree

Source: Marshall JC, Bosco L, Adhikari NK, Connolly B, Diaz JV, Dorman T, et al. What is an intensive care unit? A report of the task force of the World Federation of Societies of Intensive and Critical Care Medicine. J Crit Care. 2017;37:270-6.[36]

Table 5SB - Guide **-** steps to apply the scoring matrix

1. Assign scores:
a. For each criterion, assign scores (0, 1, or 2) based on the survey data for each ICU
2. Calculate weighted scores:
a. Multiply the scores by the corresponding weights
3. Sum total scores:
a. Add all weighted scores to obtain the total score for each ICU.
4. Determine ICU level:
a. Categorize each ICU based on thresholds:
i. Level 1: ≤ 33% of the maximum score
ii. Level 2: 34 - 66% of the maximum score
iii. Level 3: ≥ 67% of the maximum score

ICU - intensive care unit.
